# Locus of Control and Negative Cognitive Styles in Adolescence as Risk Factors for Depression Onset in Young Adulthood: Findings From a Prospective Birth Cohort Study

**DOI:** 10.3389/fpsyg.2021.599240

**Published:** 2021-03-25

**Authors:** Ilaria Costantini, Alex S. F. Kwong, Daniel Smith, Melanie Lewcock, Deborah A. Lawlor, Paul Moran, Kate Tilling, Jean Golding, Rebecca M. Pearson

**Affiliations:** ^1^Centre for Academic Mental Health at the University of Bristol, Oakfield House, Bristol, United Kingdom; ^2^Department of Experimental Psychology, School of Psychological Science, University of Bristol, Bristol, United Kingdom; ^3^Department of Psychiatry, University of Edinburgh, Edinburgh, United Kingdom; ^4^Medical Research Council Integrative Epidemiology Unit at the University of Bristol, Bristol, United Kingdom; ^5^Department of Population Health Sciences, Bristol Medical School, University of Bristol, Bristol, United Kingdom; ^6^National Institute for Health Research, Biomedical Research Centre, University Hospitals Bristol National Health Service Foundation Trust and University of Bristol, Bristol, United Kingdom; ^7^Centre for Academic Child Health, Population Health Sciences, Bristol Medical School, University of Bristol, Bristol, United Kingdom

**Keywords:** Avon Longitudinal Study of Parent and Children (ALSPAC), locus of control, negative cognitive styles, parenthood, young adulthood, depression, cohort study

## Abstract

Whilst previous observational studies have linked negative thought processes such as an external locus of control and holding negative cognitive styles with depression, the directionality of these associations and the potential role that these factors play in the transition to adulthood and parenthood has not yet been investigated. This study examined the association between locus of control and negative cognitive styles in adolescence and probable depression in young adulthood and whether parenthood moderated these associations. Using a UK prospective population-based birth cohort study: the Avon Longitudinal Study of Parents and Children (ALSPAC), we examined the association between external locus of control and negative cognitive styles in adolescence with odds of depression in 4,301 young adults using logistic regression models unadjusted and adjusted for potential confounding factors. Interaction terms were employed to examine whether parenthood (i.e., having become a parent or not) moderated these associations. Over 20% of young adults in our sample were at or above the clinical threshold indicating probable depression. For each standard deviation (SD) increase in external locus of control in adolescence, there was a 19% (95% CI: 8–32%) higher odds of having probable depression in young adulthood, after adjusting for various confounding factors including baseline mood and different demographic and life events variables. Similarly, for each SD increase in negative cognitive styles in adolescence, there was a 29% (95% CI: 16–44%) higher odds of having probable depression in the adjusted model. We found little evidence that parenthood status moderated the relationship between external locus of control or negative cognitive styles in adolescence and probable depression following adjustment for confounding factors. Effect estimates were comparable when performed in the complete case dataset. These findings suggest that having an external locus of control and holding negative cognitive styles in mid- to late adolescence is associated with an increased likelihood of probable depression in young adulthood.

## Introduction

Depression is among the most common mental health disorders, with a lifetime prevalence ranging from 10 to 20% worldwide (Kessler et al., 2005; Lim et al., 2018). The substantial personal and societal costs associated with depression make it the third largest global contributor to Years Lost due to Disability (YLD) in women and fifth largest contributor to YLD in men (James et al., 2018). The effects of depression may be especially detrimental when occurring during pregnancy and in the post-partum period because of the potential impact on the fetus and child (Bauer et al., 2014; Gelaye et al., 2016; Rebecca M Pearson et al., 2018) [e.g., through the impact of depression on parenting practices (Lovejoy et al., 2000; Wyatt Kaminski et al., 2008; Wilson and Durbin, 2010)], and on the parent [e.g., it is the leading cause of mortality in high income countries due to suicide (Johannsen et al., 2016)]. In addition to the potential impact of maternal depression on child outcomes, growing evidence supports the role of common risk factors in the development of ante- and post-partum depression for both women and men along with the detrimental consequences that these mood disruptions have on later parenting practices and on child well-being (Ramchandani and Psychogiou, 2009; Gutierrez-Galve et al., 2019; Kiviruusu et al., 2020).

Identifying modifiable risk factors in adolescence which may influence depression onset in early adulthood could assist in the development of preventative strategies to reduce depression risk. Further, understanding the moderating role of parental status in this association could help tailor specific interventions targeting this possibly higher-risk group. Whilst extensive research has been devoted to the identification of risk factors for depression across different phases of life (Cole and Dendukuri, 2003; Al-Modallal et al., 2008; Leigh and Milgrom, 2008; Lemstra et al., 2008; Côté et al., 2009; Ripke et al., 2013; Biaggi et al., 2016; Khazanov and Ruscio, 2016; Köhler et al., 2018), few studies have investigated the role of risk factors for depression during an important transition of life, such as from adolescence into young adulthood or during the transition to parenthood (Schwartz et al., 2005; Ghosh, 2017; Sawyer et al., 2018; Kathryn, 2019).

Cognitive and personality theories of depression (Abramson et al., 1997; Rubenstein et al., 2016) suggest that variation in beliefs about the world and how an individual interprets negative events around them are key factors underlying vulnerability to depression. One important aspect in an individual's outlook is a concept termed locus of control (LOC). Rotter (Rotter, 1966) first defined locus of control as “…the degree to which persons expect that a reinforcement or an outcome of their behavior is contingent on their own behavior or personal characteristics vs. the degree to which persons expect that the reinforcement or outcome is a function of chance, luck, or fate, is under the control of powerful others, or is simply unpredictable.” Attributing life events to external factors such as fate, luck, other people's power and/or considering them as unpredictable (i.e., external locus of control) has been found to be a risk factor for several adverse outcomes such as lower educational attainment (Golding et al., 2019), substance misuse (Lassi et al., 2019), chronicity of depression (Wiersma et al., 2011), non-suicidal and suicidal behaviors and ideation (Wester et al., 2016; Crandall et al., 2018), psychotic experiences (Thompson et al., 2011; Sullivan et al., 2017) and prenatal depression (Dimitrovsky et al., 1987; Richardson et al., 2012). Conversely, holding more internal locus of control beliefs has been associated with greater academic achievement (Findley and Cooper, 1983) and more positive work related feelings (Ng et al., 2006), sense of happiness (Pannells and Claxton, 2008) and lower depression (Crandall et al., 2018). However, without prospectively collected measures, these associations may be the result of reverse causation, namely depression could be causing the individual to have an external locus of control and negative cognitive styles and not vice-versa. Furthermore, residual confounding (i.e., due to unmeasured or imprecisely measured confounders) may still bias these estimates. For example, residual confounding could lead to an inflated estimate of the association due to unmeasured common causes of both our exposures and depression (e.g., if attachment insecurity caused both an external LOC and NCS and increased risk of depression).

“Negative cognitive styles” (Alloy et al., 1988) is an umbrella term that describes an individual's tendency to interpret causes of negative events as internal, global, and stable. These cognitive attributions are usually referred in the psychological literature as Negative Cognitive Schemas or Negative Cognitive Styles (NCS), as defined in Abramson's “hopelessness theory of depression” (Alloy et al., 1988). For example, after a confrontational discussion with one's partner, an individual holding NCS would attribute the cause of the negative event (i.e. the confrontational discussion) to his or her own bad character (internal), which will affect other aspects of his or her life (global), and will continue to negatively influence his or her life (stable). NCS have been associated with different negative outcomes such as depression (Nolen-Hoeksema et al., 1986; Sweeney et al., 1986; Abramson et al., 1989, 1997; Liu et al., 2015), suicidal ideation (Alloy et al., 2000) and more scholastic achievement related problems (Nolen-Hoeksema et al., 1986).

There is already evidence linking external LOC and NCS to depression (Benassi et al., 1988; Ross and Mirowsky, 1989; Kelvin et al., 1999; Evans et al., 2005; Richardson et al., 2012). Meta-analyses of observational studies found evidence for an association of LOC (Cheng et al., 2013) and NCS (Sweeney et al., 1986; Hong and Cheung, 2014) with depression. However, most studies included in these meta-analyses were cross-sectional making the potential direction of the association between LOC and depression unclear. Several theories suggest different directions of association. External LOC and NCS could be the result of current states of depression (“state hypothesis”) or a consequence of previous depression (“scar hypothesis”) (Lewinsohn et al., 1981). In support of both state and scar models, small experimental studies provide evidence that experimental manipulation of mood results in distorted cognitions (Kelvin et al., 1999) and that activation of depressed memories reduces perceived control (Obhi et al., 2013); similarly, the seminal work of Seligman on dogs demonstrated that induced lack of control led to learned helplessness, which shares various features with reactive depression (Seligman, 1972). Alternatively, NCS and LOC may represent a causal vulnerability factor for future depression as hypothesized in the vulnerability theory of depression (Barnett and Gotlib, 1988; Abramson et al., 1989). Evidence from longitudinal studies have also highlighted the role of NCS as a risk factor for future depression independently from baseline levels of depression (Nolen-Hoeksema et al., 1986; Alloy et al., 2000; Evans et al., 2005; Pearson et al., 2015) therefore representing a vulnerability factor beyond current state of depression. However, these studies only adjusted for concurrent depression and they did not investigate whether NCS could have been explained by past depression (“scar hypothesis”), leaving the question of whether these constructs have arisen in response to previous depressive symptoms unanswered. Understanding the direction of the relationship is important because NCS and LOC would only be suitable as potential prevention targets for depression if these factors lead to later depression.

Investigating how these psychological constructs (i.e., LOC and NCS) relate to onset of depression could be especially important because they are relatively stable across time (Dozois, 2007; Hankin, 2008; Elkins et al., 2017; Nowicki et al., 2018), can be modified, and effective interventions that target them are already available [e.g., cognitive behavioral therapy (CBT)] (Simmons and Parsons, 1983; Sharp et al., 1997; Osamuyi, 2000; Dozois and Quilty, 2013). Assessing the potential causal role of such constructs in adolescence may lead to implementation of these interventions at a preventive level at this developmental stage (Mehrtak et al., 2017). Moreover, understanding the role of thinking styles (locus of control and negative cognitive styles) in depression onset may help in the early identification of at-risk populations.

### The Present Study

The current study, based on participants from a large contemporary UK cohort, examined the association between both external LOC and NCS and depression in young adults and explored whether these associations were moderated by parenthood. We estimated the magnitude of these associations whilst adjusting for the effects of confounding variables related to baseline (i.e., concurrent mood) or previous mood and various socio-demographic factors and life events.

By using longitudinal data from ALSPAC, we can disentangle whether our identified risk factors are implicated in depression onset or are merely early manifestations of underlying depressive symptomatology and/or the consequences of common causes of LOC and NCS and depression. In addition, we can compare these relationships in parents and non-parents from the same original sample.

This study aimed to answer two main questions:

1. Are LOC and NCS associated with depression onset in young adults, independently from various confounding factors?
2. Does being a parent moderate the relationship between both LOC and NCS in adolescence and depression in young adulthood?

## Materials and Methods

### Participants

This study used data obtained from participants of the Avon Longitudinal Study of Parents and Children (ALSPAC), also known as Children of the 90s, which is an ongoing prospective population-based birth cohort study. The aim of ALSPAC is to investigate genetic and environmental determinants of health; for this reason, several biological, geographical, environmental, psychological and other variables were collected, including the child LOC, NCS and depressive symptoms which are used in our analyses (Golding, 2004; Boyd et al., 2013; Fraser et al., 2013). Between 1990 and 1992, all pregnant women residing in Bristol and the surrounding area, previously known as Avon county, were invited to take part in ALSPAC. 14,541 pregnant women were recruited. The original mothers and partners [Generation 0: ALSPAC-G0 (Fraser et al., 2013)], and their living children [Generation 1: ALSPAC-G1 (Boyd et al., 2013)], have been followed-up regularly since recruitment through questionnaires and clinic assessments. Complete details of the data and other information are available online and can been found at the following address (www.bris.ac.uk/alspac), and the ALSPAC data dictionary can be found at (www.bris.ac.uk/alspac/researchers/data-access/data-dictionary). Ethical approval for the study was obtained from the ALSPAC Law and Ethics Committee and South West National Health Service (NHS) Research Ethics Committee; participants gave written informed data consent.

The present study is based on ALSPAC-G1 participants who had information available on parenthood status and who had completed a self-report questionnaire on depression at 23 years of age (Short Mood and Feelings Questionnaire; *N* = 4,022) or, alternatively, who had completed the same self-report questionnaire at 21 or 22 years of age (whose information were used to impute the main outcome). Participants and researchers involved in assessments were unaware of specific hypotheses or any results of previous assessments.

**Figure 1:**
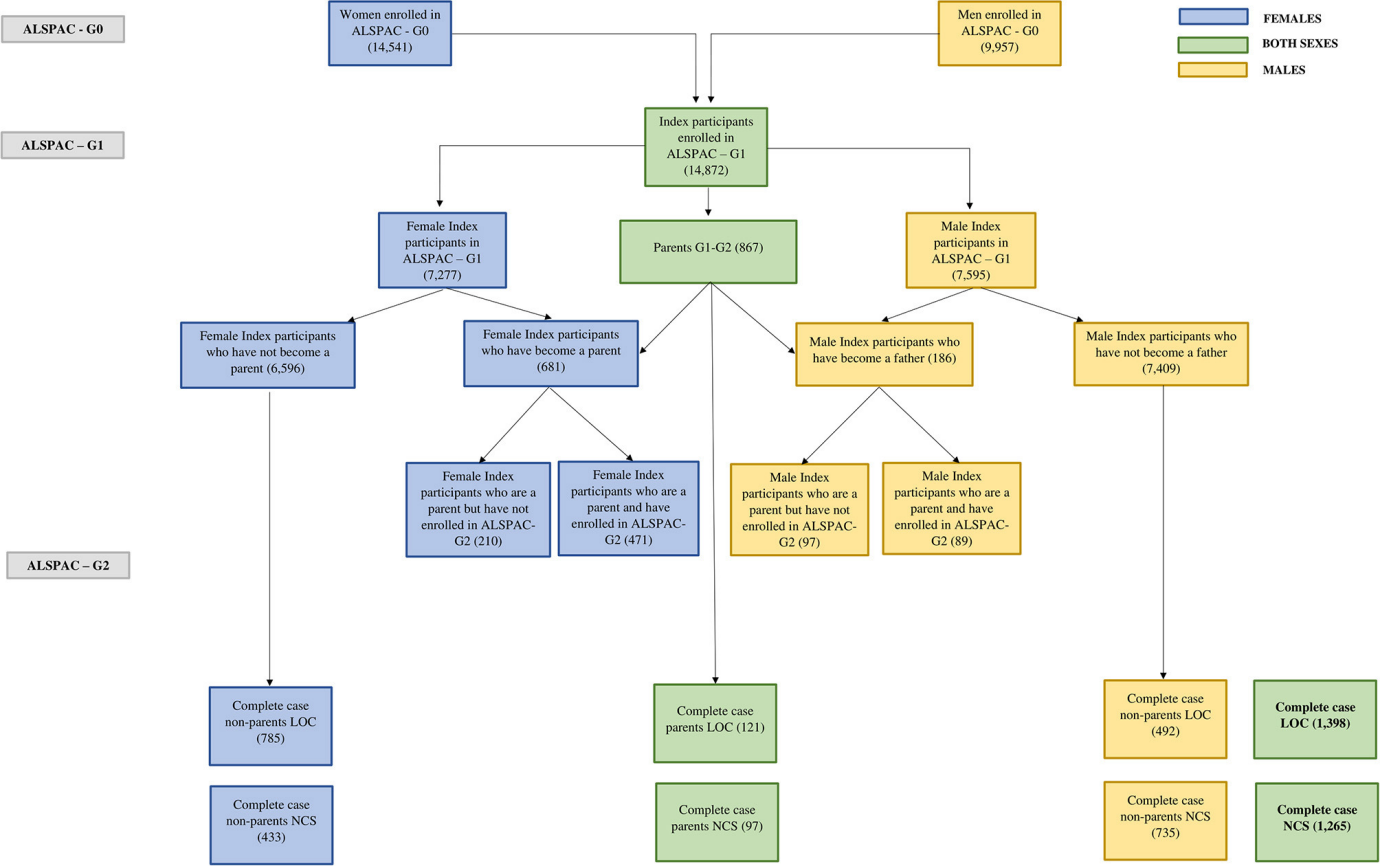
ALSPAC Flow. The present figure illustrates the sample included in our analyses (bottom rows of the graph) for both the LOC and NCS models. It describes the flow from the original mothers (ALSPAC-G0) recruited in the study up to the participants who had all the information needed to be eligible for our analysis. In the center of the figure we also illustrate how many ALSPAC-G1 participants are known to have become parents and how many have enrolled their children into the next ALSPAC generation (ALSPAC-G2). Different colors are used to distinguish between males and females. The legend of the colors is embedded in the figure.

A flow chart describes ALSPAC-G1 participants included in this analysis (Figure 1). Sample characteristics of those who contributed to the analyses and of those who did not because of missing data or loss to follow-up are reported in Supplementary Table 1.

### Measures

#### Exposure Measures: LOC and NCS

The psychological risk factors considered in G1 children/adolescents were as follows:

- LOC was assessed with the shortened version of the Children's Nowicki-Strickland Internal-External scale (CNSIE) (Nowicki and Strickland, 1973) (*N* = 5,101). A paper version of the questionnaire was sent to the homes of participants as part of the 198-month assessment. The mean age at completion of the LOC measure was at approximately 16 years and 2 months. A total score was derived by the sum of each item response with a higher score representing a more external style (Supplementary Table 2 for items). CNSIE showed a Cronbach's α of 0.57, which indicates levels of internal consistency that are slightly below satisfactory (i.e., Cronbach's α > 0.60). However, this measure has been used in previous research research using the same sample as our analysis (Culpin et al., 2015) and does not largely deviate from the estimates presented by Nowicki (2018) who reported internal consistency estimates ranging from 0.60 to 0.70 for locus of control as measured with the CNSIE. Analyses were performed using the continuous and standardized z-score.
- NCS was assessed with the Cognitive Style Questionnaire Short Form (CSQ-SF) (Meins et al., 2012), which was administered to the ALSPAC index child (*N* = 4,171) by an interviewer who collected the responses on paper at the Fourth Teen Focus research clinic when participants were an average age of 17 years and 10 months. The questionnaire presents different hypothetical scenarios and asks the participant to imagine those scenarios happening to them and to rate their reactions to those situations (Supplementary Table 3 for items). Four possible patterns of causal attributions are derived (internal, stable, global, self-worth). Possible total scores range from 64 to 320, with higher scores indicating a more negative cognitive style. The total score for CSQ-SF demonstrated a good internal consistency, with a Cronbach's α of 0.88. Analyses were performed using standardized total z-score after median imputation.

#### Outcome Measure: Probable Depression Diagnosis

The short mood and feelings questionnaire (SMFQ) (Ancold and Stephen, 1995; Turner et al., 2014) was used to measure depressive symptoms in ALSPAC-G1 participants (*N* = 4,022). The SMFQ is a 13-item questionnaire that measures depressive symptomatology over the preceding 2 weeks with higher scores indicating more severe depressive symptoms (range 0–26) (Supplementary Table 4 for items). The SMFQ demonstrated good discriminatory abilities for identification of depression as measured by Computerized Interview Schedule-Revised (CIS-R) which uses ICD−10 diagnostic criteria (area under ROC curve = 0.90) (Turner et al., 2014). The SMFQ was administered when the participants were approximately 23 years of age [mean age of SMFQ completion = 23.9 years old (SD = 0.5)]. The questionnaire was available to complete in either online or paper format; responses to the online questionnaires were collected and managed using REDCap electronic data capture tools (Harris et al., 2009) hosted at the University of Bristol. £10 shopping vouchers were sent to all the participants who took part in the study. High levels of depression were defined by a score of 11 or higher according to commonly used clinical thresholds (Turner et al., 2014; Kwong et al., 2019). In this study, analysis of the internal consistency of the SMFQ using Cronbach's α showed good levels of inter-item reliability (α = 0.91). The SMFQ was used to assess depression symptomatology in all ALSPAC-G1 participants (regardless of their parental status). However, because of the availability of questions on parental status, we were able to discern levels of depression in participants who had become parents. Participants who had become parents or expectant parents after the SMFQ assessment were not included in the analyses.

#### Moderating Variable: Parental Status

The variable indicating parental status was built including all ALSPAC participants who were known to have become parents or were expecting a child at the time of the assessment of the outcome (SMFQ at 23 years of age) (*N* = 790). This information was either obtained by including all participants who enrolled as parents of the second generation of ALSPAC (ALSPAC-G2) (Lawlor et al., 2019) or by including those ALSPAC participants who had responded affirmatively (i.e., they have a child) to at least one of the repeated questions about parenting (from age 16 to 24). The questions that have been used to identify parents are reported in the Supplementary Table 5.

### Confounding Factors

**Figure 2:**
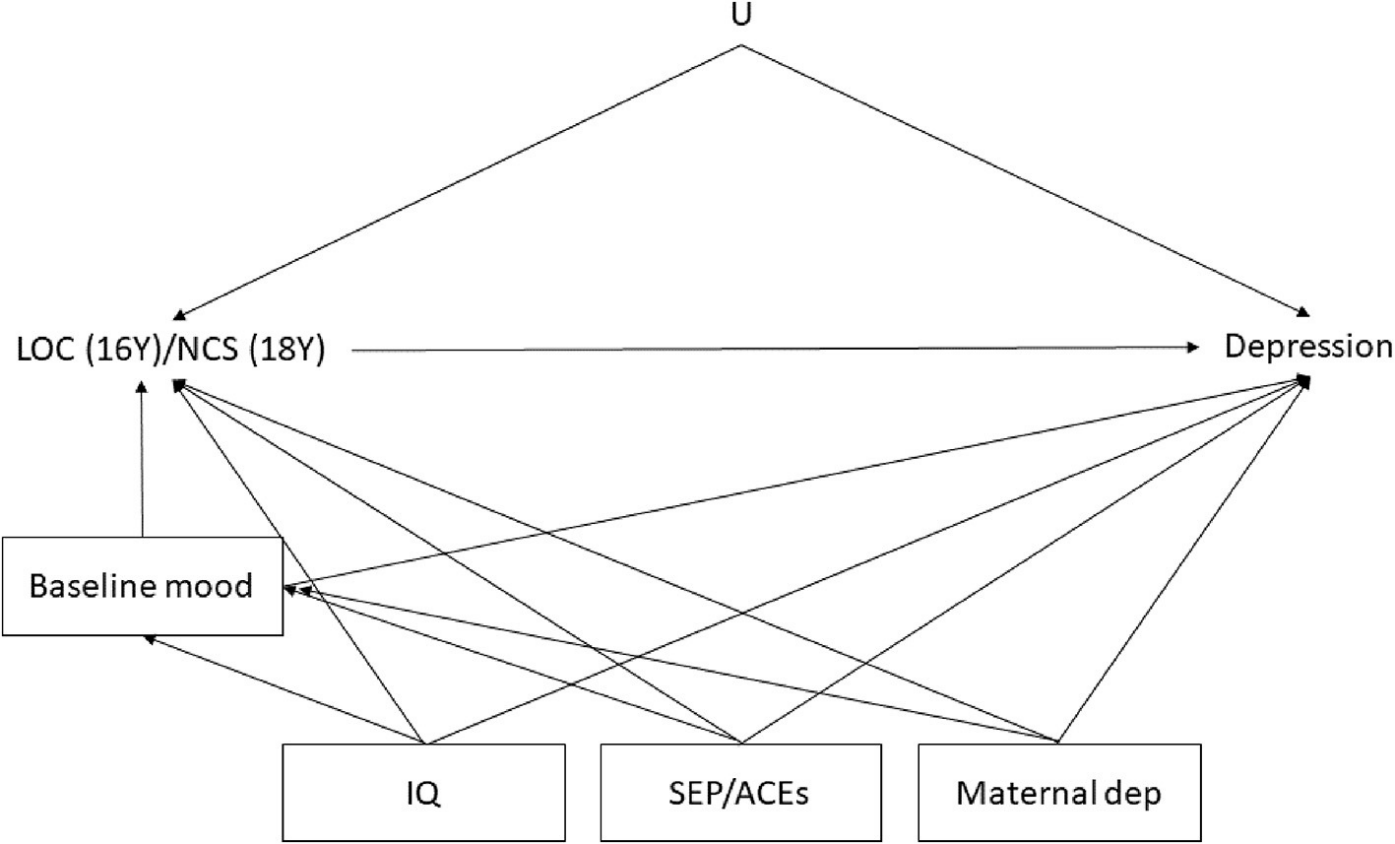
Directed Acyclic Graph (DAG) representing hypothesized causal relationship between our exposures (locus of control and negative cognitive styles) and our outcome (depression). “U” represents the unmeasured/unknown confounding factors which we cannot adjust for. In the Locus of control model, baseline depression and anxiety were measured with the SMFQ and the GAD subscale of DAWBA, respectively. In the Negative Cognitive Styles model, baseline depression and anxiety were measured with depression and anxiety subscale of CIS-R at 18 years old. Depression represents the outcomes which was measured with the SMFQ at 23 years of age. IQ was measured at 8 years old as a total score derived by the Wechsler Intelligence Scale for Children, Socio Economic Position related variables included: maternal education, maternal social class and a continuous score of the Adverse Childhood Experiences (ACE) variables, sex. “Maternal Dep” stands for the maternal (ALSPAC-G0) depression during pregnancy measured with the EPDS. Correlations between confounding factors are not represented for simplicity.

Being more externally oriented or holding negative cognitive styles whilst reflecting upon negative causes of events may be the result of an underlying depressive disorder, which is also a cause of future depression, and thus could act as a confounding variable. Therefore, we adjusted for baseline (concurrent) mood both in the LOC and NCS models. Additionally, as a sensitivity analysis we adjusted for depression symptoms measured in the previous visit instead of the concurrent visit (in separate models for LOC and NCS and using a measure of depressive symptoms at 13 and 16 years of age, respectively). Adjustment for depression during the previous visit was performed because a prior experience of depression could have still acted as a common cause of both LOC and NCS and subsequent depression even if depression symptomatology was absent at the moment in which LOC and NCS were assessed (“scar hypothesis”). Other confounding factors adjusted in the models were: anxiety symptoms, sex (males vs. females) (Dekker et al., 2007; Sterba et al., 2007; Kuehner, 2017), variables related to socio-economic positions (i.e., maternal education and occupational social class) (Stoolmiller et al., 2005; Costello et al., 2008), adverse experiences during childhood (e.g., physical, sexual abuse, bullying and parental separation) (Stoolmiller et al., 2005; Singham et al., 2017), lower cognitive abilities (Petersen et al., 1993) and maternal depression (Petersen et al., 1993; Rebecca M Pearson et al., 2013) (Figure 2). A complete list of covariate variables that have been used is provided in Supplementary Table 6.

### Statistical Analyses

All analyses were performed with Stata (version 15.1). We employed unadjusted and adjusted logistic regressions to examine the associations between both LOC and NCS and depression. Primary analyses were conducted on the imputed datasets. To address the first question of this study (i.e., “are LOC and NCS associated with later probable depression independently of known confounding factors?”) we employed multivariable logistic regression with continuous risk factors (i.e., LOC and NCS) which were standardized to create Z scores with a mean of 0 and a SD of 1 adjusted for the above-mentioned list of confounding factors. We used commonly used thresholds to define elevated probable depression which have been validated against ICD-10 depression via clinical interviews. Odds Ratios (OR) are presented in order to clearly illustrate how our risk factors are associated with clinically relevant depressive symptoms. Moreover, by using logistic models we overcome the non-normal distribution of the continuous outcome which has a skewed distribution in this sample (Kwong, 2019). Analyses were stratified by sex to investigate the strength of these associations in males and females. This was conducted because there are higher rates of depression in females than males (Kuehner, 2017). To address the second question of this study (i.e., “Does being a parent moderate the relationship between both LOC and NCS in adolescence and depression in young adulthood?”), moderation analyses where conducted to explore a potential interaction between parental status and our exposures (i.e., LOC and NCS) for depression at 23 years, given a slightly higher prevalence of depression in parents compared to non-parents in our sample.

95% Confidence Intervals (CIs) were reported for all analyses. Distributions of demographic and psychological risk factors across the two groups (parents and non-parents) were compared through chi-squared tests (categorical variables) and Welch un-paired *t*-tests (continuous variables) (Delacre et al., 2017). We also present analyses on those with no missing data (known as complete case analyses) in the Supplementary Materials.

### Missing Data

Sample attrition led to a substantial amount of missing data, particularly for the outcome and the confounders. After analyzing proportions of missingness according to different socio-demographic indicators and exploring variables associated to missingness (Supplementary Tables 7 and 8) we performed analysis under the assumption that data were missing at random (MAR). MAR refers to a type of missingness that can be explained by observed variables (e.g., socioeconomic status, previous measures of mental health). Since some of the variables that are associated with missingness (i.e., auxiliary variables in our imputation model) were not included as confounding factors, using the complete case dataset may provide biased estimates (Hughes et al., 2019). For these reasons, our primary analyses are performed in the multiply imputed datasets. Thanks to the wealth of auxiliary variables (i.e., variables associated with missingness and with the variables in the model) that we were able to include in our imputation model the imputed datasets should increase statistical power (Sterne et al., 2009; Hughes et al., 2019). As the underlying assumptions differ between complete case and multiple imputation analyses, consistent results with both methods increase confidence that they are not biased by missing data (Hughes et al., 2019). Multiple imputation through chained equations, also known as fully conditional specification, on 100 imputed datasets with 50 cycles of regression switching were performed separately for parents and non-parents (Tilling et al., 2016) using the ice command in Stata 15.1 (Royston, 2005, 2007; Sterne et al., 2009). We used predictive mean matching (STATA match command) to match the imputed exposures and outcome variables on the distribution of the complete case variables (Rubin, 1986). When imputing, we included all the variables of our models (including covariates) and auxiliary variables which were associated with the outcome (depression) and/or with the missingness (a list of variables used is presented in the Supplementary Table 9). Parents and non-parents imputed datasets were appended after imputations using the mi append, automatic command. Including auxiliary variables is recommended to make the MAR assumption more plausible (Hughes et al., 2019), however we kept the number of covariates included relatively small to avoid overfitting the models (Lee et al., 2020). Estimates from different imputed datasets were combined and analyzed using Rubin's rules (White et al., 2011). Monte Carlo errors were <10% of the standard error and fraction of missing information (FMI) values were no larger than 0.53, both indicating an adequate level of statistical reproducibility of the multiply imputed analyses (Madley-Dowd et al., 2019).

## Results

### Demographic Differences Between Parents and Non-parents

Participants who had become parents and those who had not were compared on basic demographic and psychological variables using a Welch *t*-test where continuous and chi-squared test where categorical (Table 1). Parents differed from their peers who had not become parents on basic socio-demographic variables and on LOC at 8 and 16 years old. Those who had become parents generally came from poorer socio-economic backgrounds (e.g., lower parental education, lower parental social class and lower income) and were more externally oriented both at 8 and 16 years of age.

**Table 1:** Demographic and psychological differences between ALPSAC-G1 parents (either those who enrolled and those who did not enroll in ALSPAC-G2) and non-parents.

**Variables^a^**	**Sample**						**Test**
	**ALSPAC-G1 Non-parents**			**ALSPAC-G1 Parents**			
	***N***	**Mean (SD)**	**95% CI**	***N***	**Mean (SD)**	**95% CI**	**Welch *t*-test**
**G0 maternal weekly income during pregnancy**	9,363	313.3 (139.4)	310.46–316.11	616	269.52 (124.04)	259.71–279.34	8.41
**Age of menarche**	3,666	12.64 (1.16)	12.61–12.68	479	12.49 (1.17)	12.39–12.59	2.65
**Locus of control 8y**	5,927	5.95 (2.08)	5.90–6.00	446	6.47 (2.01)	6.28–6.66	−5.25
**Locus of control 16y**	4,645	3.08 (2.08)	3.02–3.14	452	4.00 (2.28)	3.79–4.21)	−8.31
**Cognitive styles 18y**	3,822	161.76 (20.07)	161.12–162.40	349	161.12 (21.32)	158.88–163.37	0.54
	***N***	**Frequencies %**		***N***	**Frequencies %**		**Chi**^**2**^
**G0 maternal education**							
Low (Lower than O level)	3,508	29.8		240	35.4		
Medium (O level)	4,050	34.4		267	39.4		32.07
High (A level or higher)	4,229	35.9		171	25.2		
**G0 Paternal education**							
Low (Lower than O level)	3,870	34.2		272	41.9		
Medium (O level)	2,399	21.2		152	23.4		25.67
High (A level or higher)	5,065	44.7		226	34.8		
**G0 Maternal social class following Office of Population Censuses and Surveys (OPCS) codes**							
Low	5,966	62.4		362	68.2		7.28
High	3,602	37.7		169	31.8		
**G0 Paternal social class following (OPCS) codes**							
Low	5,667	54.5		384	65.2		25.94
High	4,738	45.5		205	34.8		
**G0 maternal depression**							
No	9,871	86.3		539	84.2		2.12
Yes	1,572	13.7		101	15.8		

*Legend: Age of menarche was measured as a derived variable where the first reported age at onset of menarche was used, Locus of control was measured at 8 and 16 years old with the CNSIE, negative cognitive styles was measured at 18 with the CSQ. Variables preceded by “G0” refer to measures which were captured in the parents of our target sample (i.e., ALSPAC-G1). Maternal and Paternal educational attainment was coded as 0 = A level or higher, 1 = O level, 2 = < O level, maternal and paternal social class was coded as 0 = high social class and 1 = low social class, G0 maternal depression refers to depression symptomatology of women during pregnancy as measured with the EPDS and dichotomised using a cut off of >12 to determine probable diagnosis of major depression*.

### Depression Levels

In the total sample, 994 (24.7%) out of 4,022 participants who completed the SMFQ at 23 years old scored at or above the clinical threshold for depression (cut-off of ≥11). When stratifying by sex, 723 (27.4%) females, and 269 males (19.5%) scored at or above the clinical threshold indicating probable depression. When stratifying by parenthood status, 851 (24.4%) non-parents and 141 (26.3%) parents scored at or above the clinical threshold for depression. Of these 851 non-parents, 248 (19.4%) were men, whereas 603 (27.4%) were women. Similarly, of the 141 parents who scored above the clinical cut-off for probable depression, 21 (20.6%) were men, whereas 120 (27.6%) were women.

Overall, depression levels were high in the complete sample irrespective of parenthood status. However, as expected, women had higher levels of depressions than men, regardless of parenthood status.

### Comparison of Characteristics Between Samples With Missing and Non-missing Information

Analysis of the socio-demographic differences across various samples indicated that the participants with missing data comprised individuals who were more disadvantaged (i.e., had lower education and more socioeconomic disadvantage) compared to those who were not lost to attrition (Supplementary Table 1). Those with complete data were more likely to come from families of parents who were educated beyond secondary school (A level or higher) and of a higher socio-economic class (e.g., non-manual jobs), to have mothers who gave birth at a later age, and had mothers who were less likely to have smoked during the first trimester of pregnancy. However, the study sample also comprised a higher percentage of participants who reported physical or sexual abuse during childhood. There was evidence that participants with missing data on depressive symptoms were more likely to have reported an external LOC at 16 years of age, had their mother reporting smoking during pregnancy, had a family which scored lower on various socioeconomic indicators and were more likely to have had a physical illness during their childhood.

### Associations Between LOC and NCS in Adolescence With Probable Depression in Adulthood

There was evidence for an association between LOC at 16 years and depression at 23 years (Table 2). An increase of one standard deviation in LOC score at 16 years was associated with a 62% (CI: 49 to 76%) increase in odds of probable depression at 23 years. After adjusting for confounding variables there was a marked attenuation toward the null, although evidence for an association between LOC and depression remained (19%, CI: 8 to 32%). Similarly, we found evidence for an association between NCS at 18 years and probable depression at 23, with a 50% increase in odds of depression for every SD increase in NCS (CI: 37 to 65%). Once adjusted for potential confounding factors the association attenuated but remained consistent (29%, CI: 16 to 44%). In addition, when using previous depression measure instead of baseline depression the results did not markedly change both in the LOC model and in the NCS model.

**Table 2:** Main analyses: unadjusted and adjusted logistic regressions of locus of control and negative cognitive styles on depression, in total sample and in parents in multiple imputed datasets.

	**Locus of control**							**Negative cognitive styles**						
**Sample^1^**	**Unadjusted^a^**			**Adjusted^b^**		**Adjusted^c^**		**Unadjusted^a^**			**Adjusted^b^**		**Adjusted^c^**	
	***N***	**OR (95% CI)**	***P*-value**	**OR (95% CI)**	***P*-value**	**OR (95% CI)**	***P*-value**	***N***	**OR (95% CI)**	***P*-value**	**OR (95% CI)**	***P*-value**	**OR (95% CI)**	***P*-value**
Total sample^c^	4,301	1.62 (1.49–1.76)	<0.001	1.19 (1.08–1.32)	0.001	1.35 (1.23–1.49)	<0.001	4,301	1.50 (1.37–1.65)	<0.001	1.29 (1.16–1.44)	<0.001	1.24 (1.11–1.39)	<0.001
Parents	509	1.82 (1.43–2.22)	<0.001	1.50 (1.10–2.04)	0.01	1.59 (1.19–2.14)	0.002	509	1.56 (1.21–2.00)	0.001	1.36 (.99–1.85)	0.05	1.30 (.95–1.79)	0.10

1 *OR, odds ratio; CI, Confidence Interval; SMFQ, Short Mood and Feeling Questionnaire*.

a *Univariable associations between standardized continuous score of LOC/NCS*.

b *Multivariable associations between standardized continuous score of LOC/NCS, confounding variables adjusted for: baseline/concurrent depression and anxiety, gender, maternal depression, maternal social class, maternal education, ACE classic total score, IQ at 8 years old*.

c *Multivariable regressions between standardized continuous score of LOC/NCS, confounding variables adjusted for: previous depression, anxiety, gender, maternal depression, maternal social class, maternal education, ACE classic total score, IQ at 8 years old*.

Finally, in the analyses performed in the complete case dataset, effect estimates were comparable to those obtained in the imputed datasets (Supplementary Tables 10–14). However, as expected, confidence intervals in the imputed analyses were narrower, indicating higher statistical precision and power.

### Parental Status as Weak Moderator of the Association Between LOC and NCS With Probable Depression

We stratified our analyses based on sex and on parenthood. Moreover, we explored whether there is an interaction between parenthood and both LOC and NCS, with depression as the outcome (Table 3). Stratification by sex did not show any strong differences in the associations between both LOC and NCS with depression in males and females (Table 4). However, when we stratified our analyses based on parental status, differences in effect estimates in parents and non-parents provided suggestive evidence for a possible moderating effect of parenthood. Thus, we investigated whether there was evidence for an interaction between parental status and both our exposures (i.e., LOC and NCS), with our outcome of interest (i.e., depression as measured by the SMFQ at 23 years). We found limited evidence for an interaction between parental status and our exposures for depression (OR: 1.10, CI: 0.94–1.30, OR: 1.14, CI: 1.00–1.29, respectively in LOC and NCS models) once adjusted for covariates.

**Table 3:** Unadjusted and adjusted odds ratio for adult depression according to continuous scores of locus of control and negative cognitive styles and stratified by parental status in multiple imputed datasets.

	**Moderation model by parenthood**							
	**Entire sample (4,301)**		**Parents^a^ (509)**		**Non-parents (3,792)**		**Interaction term**	
	**OR**	**95% CI, p**	**OR**	**95% CI, p**	**OR**	**95% CI, p**	**OR**	**95% CI, p**
Locus of control	1.62	1.49–1.76, <0.001	1.82	1.43–2.22, <0.001	1.61	1.47–1.76, <0.001	1.10	0.95–1.28, 0.22
Locus of control adjusted for confounding factors^b^	1.19	1.08–1.32, 0.001	1.50	1.10–2.04, 0.01	1.17	1.06–1.31, 0.003	1.10	0.94–1.30, 0.23
Cognitive styles	1.50	1.37–1.65, <0.001	1.56	1.21–2.00, 0.001	1.49	1.35–1.65, <0.001	1.22	1.07–1.40, 0.004
Cognitive styles adjusted for confounding factors^b^	1.29	1.16–1.44, <0.001	1.36	.99–1.85, 0.05	1.29	1.15–1.44, <0.001	1.18	1.03–1.34, 0.01

*Outcome: binary SMFQ at 23 years*.

a *All ALSPAC-G1 participants who have become parents (regardless their enrolment in ALSPAC-G2)*.

b *Multivariable regressions between standardized continuous score of LOC/NCS and depression, adjusted for the following confounding variables adjusted for: baseline depression and anxiety, gender, maternal depression, maternal social class, maternal education, ACE classic total score, IQ at 8 years old*.

**Table 4:** Unadjusted and adjusted odds ratio for adult depression according to continuous scores of locus of control and negative cognitive styles and stratified by sex in multiple imputed datasets.

	**Entire sample (4,301)**		**Male (1,541)**		**Female (2,760)**	
	**OR**	**95% CI, p**	**OR**	**95% CI, p**	**OR**	**95% CI, p**
Locus of control	1.62	1.49–1.76, <0.001	1.53	1.31–1.79, < .001	1.62	1.47–1.78, <0.001
Locus of control adjusted for confounding factors^a^	1.19	1.08–1.32, 0.001	1.16	0.96–1.40, 0.13	1.21	1.07–1.36, 0.002
Cognitive styles	1.50	1.37–1.65, <0.001	1.44	1.22–1.71, <0.001	1.51	1.36–1.68, <0.001
Cognitive styles adjusted for confounding factors^a^	1.29	1.16–1.44, <0.001	1.27	1.05–1.53, 0.01	1.30	1.16–1.46, <0.001

Outcome: binary SMFQ at 23 years

a *Multivariable regressions between standardized continuous score of LOC/NCS and depression, adjusted for the following confounding variables adjusted for: baseline depression and anxiety, gender, maternal depression, maternal social class, maternal education, ACE classic total score, IQ at 8 years old*.

## Discussion

The present study, based on a large prospective population-based cohort, provides further evidence for a prospective association between both LOC and NCS and probable diagnoses of depression in young adults. Moreover, this study provides the first evidence on whether parenthood has a moderating role on these associations. As expected, adolescents who reported having a more external LOC and had cognitive biases in the attribution of causality for negative events (i.e., they held global, stable and internal self-blaming beliefs about these events; NCS) were more likely to have higher levels of probable depression in young adulthood. Although the effect estimates of LOC and NCS on depression attenuated somewhat after adjusting for various confounding factors, there was still evidence to support their independent association with probable depression and, thus, sustaining the vulnerability theory (Abramson et al., 1999) compared to the “state” and “scar” hypothesis (Lewinsohn et al., 1981). In fact, by adjusting for depression and anxiety symptoms measured both prior to and at the time of the assessment of the exposure, we were able to exclude the possibility that all the observed association between LOC and NCS with later depression was driven from the current mood state (“state hypothesis”) and previous mood (“scar hypothesis”). In addition, even small effect sizes of risk factors measured as continuous variables may have an important effect at the population level when the outcome studied is common. For example, if we assume that our findings are valid, a 1 SD increase in ELOC (adjusted model) would lead to a 4% higher absolute risk for probable depression in young adults (28 vs. 24%) given the relative risk of probable depression (OR=1.19 per SD increase in ELOC) and prevalence of this condition in our sample (24%). This would mean that, if our findings are generalisable to the geographical area surrounding Bristol (i.e., South West England), which has a population of 63,929 individuals who were 24 years of age (OfN, 2018), a 1 SD increase in ELOC during adolescence would translate to 2,915 more people at risk of probable depression (18,258 vs. 15,343 individuals). Similarly, a 1 SD increase in NCS (adjusted model) would lead to a 7% higher absolute risk for probable depression in young adults (31 vs. 24%) given the relative risk of probable depression (OR of 1.29 per SD increase in NCS). This would translate into an additional 4,475 more people at risk of probable depression (19,818 vs. 15, 343 individuals). These figures highlight the potential public health utility in targeting risk factors when the outcome of interest (i.e., depression) is common in the population.

One novel aspect of the current contribution is the age range that we have explored from mid- to late adolescence to early adulthood and the fact that we have explored the probability of depression in young adults who have become parents and in those who have not. If the relationship between external LOC/NCS and later depression was found to be causal (e.g., through a well-conducted randomized controlled trial), developing preventive interventions to enhance an internal sense of control and reduce automatic negative biases could lower individuals' risk of developing depressive symptomatology. For example, when negative events occur such an intervention could increase the level of resilience an individual holds, providing positive resources to young adults to use in the presence of changes (e.g., transition to adulthood or parenthood) and in the presence of adverse events (e.g., COVID-19 outbreak) (Ng-Knight and Schoon, 2017). Indeed, holding an internal sense of control may be a particularly important resource in the transition to adulthood, when important individuation processes (e.g., choosing a university, starting a job, moving out of one's parent's house, starting a family) are occurring (Mirowsky and Ross, 2007; Settersten, 2007; Surjadi et al., 2011). This may be especially true in individualistic societies where values like individual sense of agency and personal responsibilities are emphasized and therefore may not be generalisable to societies with different values systems (Cheng et al., 2013).

### Is Being a Parent a Risk Factor?

One aim of this study was to explore whether becoming a parent moderated the association between LOC and NCS in adolescence and liability to depression in young adulthood. In this cohort, becoming a parent at a young age does not seem to represent a strong risk factor for depression development. Prevalence of depression is high in both groups (parents and non-parents) consistent with what has previously been reported in the literature (Evenson and Simon, 2005; Rimehaug and Wallander, 2010). In this study we have combined mothers and fathers into a single sample of parents because we lack statistical power to detect differential associations between our risk factors and our outcomes across groups stratified on both sex and parenthood status, given low numbers of fathers (*N* = 19 in the fully adjusted model). However, being a parent represents a shift from being largely only responsible for oneself to being responsible for dependents, independent of the sex of the parent. It is this shift in responsibility that is hypothesized to modify the relationship between adolescent psychological risk factors and probable depression. By stratifying the analyses according to parenthood status, at best we found weak evidence that parenthood modified the strength of the association, with effect estimates and confidence intervals largely overlapping. This is a topical question because, whereas substantial evidence exists supporting the gender gap in prevalence estimates for specific disorders (Kuehner, 2003, 2017) [mainly internalizing vs. externalizing disorders, where the former disorders are generally more common in females (Kuehner, 2017)], there is less evidence to support the possible moderating role of a key event in many people's lives: transitioning to parenthood. One previous study (Lewis et al., 1999) explored how perceived control changes from adolescence to adulthood and how pregnancy in women may reduce or strengthen the association between adolescent LOC and adulthood LOC but did not consider its impact on depression liability. The authors found having a more internal LOC was positively associated with age and that dropping out of school, but not getting pregnant, was negatively associated with developing a more internal LOC (Lewis et al., 1999). In fact, whereas the authors found cross-sectionally that pregnant women or men with pregnant partners felt less in control of their lives, becoming a parent did not moderate the observed increase in internality of locus of control with age. However, when transition to parenthood was accompanied by dropping out of school, the magnitude of the previously observed increase in locus of control with age was reduced. Transition to fatherhood has been under-investigated compared to the transition to motherhood (Recto and Champion, 2020). However, some research has identified dysfunctional cognition and external locus of control as risk factors for the development of depressive symptoms during this transition to fatherhood (Keeton et al., 2008; Sockol and Allred, 2018). Holding dysfunctional cognitions and beliefs may be especially relevant during the transition to parenthood, due to the shift in demands from one's own needs to looking after a dependent child. This new role and responsibility may amplify the impact of negative cognitions linked to low self-worth on mood. These findings seem to preliminarily suggest that intervening on adolescent's LOC and NCS may be beneficial in adolescents transitioning to adulthood, regardless of their parental status, in reducing risk of probable depression later in life. This could potentially have broad societal consequences as depression is an increasingly common mental health problems, affecting a large number of individuals (World Health Organization, 2017). Developing preventative interventions that target LOC and NCS to reduce risk of depression could be warranted if a well-conducted RCT were to confirm our results (see Box 1 for case example).

Box 1 **Case example.**

XXX is a 16-year-old student who is preparing for his General Certificate of Secondary Education (GCSE). He is experiencing a lot of anxiety and uncertainty about how the exams might go and he is having difficulties in concentrating. He took part in a Cognitive Behavioral Therapy course where his therapist identified a series of conditional beliefs: “Nothing I do can change how the exams will go, it is just a matter of luck!.” He would often feel guilty and stressed about the upcoming exams and in study groups he would often lose focus while studying. In one mock exam offered by the school, he had forgotten to complete a math problem. He then thought “All my exams will go terribly” and “I am such an idiot and I can't learn anything” and his mood started to worsen. The therapist helped him in re-assessing the automatic thoughts that XXX had after that minor negative event, in turn helping him to reduce feelings of low self-worth and changing those generalized beliefs into more specific ones. Rather than thinking that passing the exams was a matter of luck, he was encouraged to feel more in control of behaviors that he could employ to improve his results (such as revising and asking teachers for help) and to realize exams are just one aspect of his life which helps him to not catastrophise from that single experience.

### Strengths and Limitations

There are several strengths of this study. To our knowledge, this is the largest study that has been conducted to date that has prospectively examined the association between LOC and NCS in adolescence and probable depressive disorder in young adulthood and is the first study to examine these associations in the transition to adulthood and parenthood. Other strengths of this study include the relatively large sample and long-term follow up as compared to previous studies (Richardson et al., 2012), the use of serial measures, and the availability of several important confounding and moderating variables.

However, there are several limitations of this study which should be acknowledged. First, the proportions of missingness were high. Around 68% (537 out of 790) of parents and 24.5% (3,482 out of 14,252) of non-parents or not known parents completed the SMFQ at 23 years of age. This may have biased our results in several ways: first, statistical power was reduced in analyses among parents which resulted in an increased imprecision around our effect estimates (i.e., wider confidence intervals); second, missingness may have introduced differential selection bias in parents compared to non-parents. For example, we may have underestimated depression in parents because the most severe cases may have been less likely to attend a clinic visit. Conversely, we may have overestimated depression in the non-parents because the least vulnerable participants may have been more likely to have moved out of the Bristol area, for example, to study elsewhere, and therefore were more likely to have dropped out or to not have attended clinic visits. Another limitation is that the use of multiple imputation assumes data is MAR, when in fact variables could be missing not at random (MNAR), meaning that missingness may have depended on unobserved data (e.g., depression outcome is missing because participants with depression did not show up to the visit). This could have biased our findings to a larger extent than those obtained from analyses restricted to the complete case dataset (Hughes et al., 2019). Since it is impossible to test whether data are MNAR without relying on further assumptions (Hughes et al., 2019), we cannot be confident in excluding this possibility as the missing data could depend on unmeasured variables. Fourth, moderation analyses often lack statistical power, therefore, to truly understand whether parenthood moderated these associations a larger sample should be employed. Fifth, as in all observational studies, we cannot exclude the possibility of residual confounding due to unmeasured or imprecisely measured confounders in our analyses. One possible solution to minimize the potential role of unmeasured confounders would be to attempt to replicate our analyses in another longitudinal cohort with a known different confounding structure (Richmond et al., 2014). Sixth, we cannot exclude the presence of measurement error. Indeed, the below satisfactory levels of internal consistency [Cronbach's α of 0.57 vs. satisfactory Cronbach's α of 0.60 (Nowicki, 2018)] indicate measurement error in LOC as assessed with the CNSIE at 16 years of age. However, we expect this measurement error to be non-differential and it is therefore likely to represent an underestimate of the true effect. Finally, we cannot exclude the possibility of chance findings and this likelihood increases with multiple hypotheses being tested. We conducted multiple tests in these analyses, however only four tests constituted primary analyses (the remaining tests were sensitivity analyses performed to examine the robustness of our findings). We did not employ corrections for multiple testing as this could be overly conservative because of non-independence of our hypotheses examined. Furthermore, we do not make categorical interpretations based on *p*-values but rather report the overall patterns of findings and strength of evidence.

In conclusion, replication of our findings in larger and more diverse samples and using different methods (e.g., RCTs) is required to refine the understanding of the evidence around the causal role of LOC and NCS in depression onset. In particular, having a large sample (e.g., with sufficient numbers of both fathers and mothers in the sample) would allow differential investigation of the relationship between LOC and NCS with probable depression in mothers and fathers separately.

### Implications

Adolescents are a key risk group and current global target for supportive interventions. Adolescence provides a privileged time-window opportunity for preventive interventions because, thanks to universal schooling, there is the possibility of early and associated screening and opportunity of receiving psychological support. This study suggests that interventions targeting LOC and NCS in adolescents could prevent young adulthood depression in parents, expectant parents and non-parents.

## Data Availability Statement

The data analyzed in this study is subject to the following licenses/restrictions: ALSPAC datasets can be accessed pending approval of research proposals. Requests to access these datasets should be directed to alspac-data@bristol.ac.uk, bbl-info@bristol.ac.uk.

## Ethics Statement

The studies involving human participants were reviewed and approved by Ethical approval for the study was obtained from the ALSPAC Law and Ethics Committee and South West National Health Service (NHS) Research Ethics Committee; participants gave written informed data consent. Written informed consent to participate in this study was provided by the participants' legal guardian/next of kin.

## Author Contributions

JG established the ALSPAC study and managed data collection, including for several of the variables used here. RP and IC contributed to conception and design of the study. ML and DL managed data collection in the ALSPAC-G2 study. IC performed the statistical analysis and wrote the first draft of the manuscript. All authors contributed to manuscript revision, read, and approved the submitted version.

## Conflict of Interest

DL reports support from Roche Diagnostics and Medtronic Ltd for research unrelated to that presented here. The remaining authors declare that the research was conducted in the absence of any commercial or financial relationships that could be construed as a potential conflict of interest.
