# Supplementary material for: Locus of Control and Negative Cognitive Styles in Adolescence as Risk Factors for Depression Onset in Young Adulthood: Findings From a Prospective Birth Cohort Study
Source: Front Psychol. 2021 Mar 25;12:599240. doi: 10.3389/fpsyg.2021.599240 (PMC8080877; doi:10.3389/fpsyg.2021.599240)
Supplement: Supplementary file 1 [file Table_1.docx]

**Supplementary Materials**

Supplementary Table 1. Distribution of socioeconomic, maternal, paternal, familial indicators and child psychosocial indicators in the original ALSPAC cohort in various sub-samples used in the study

|  | Total ALSPAC-G1 sample (N=14,872) % | Sample with LOC measured at 16 y (N= 5,106) % | Sample with NCS measured at 18 y (N= 4,173) % | Sample with complete response on SMFQ at 23 y (N= 4,022) % | Full sample LOC and SMFQ (N=2,921) % | Full sample NCS and SMFQ (N=2,379) % | Complete sample LOC +confounding var (N=1,398)  % | Complete sample NCS +confounding var (N=1,265)  % |
| --- | --- | --- | --- | --- | --- | --- | --- | --- |
| **Maternal education** |  |  |  |  |  |  |  |  |
| A level or higher | 35.4 | 48.0 | 48.5 | 47.2 | 50.9 | 52.0 | 56.6 | 56.9 |
| O level | 34.6 | 33.3 | 33.7 | 34.5 | 33.8 | 33.3 | 32.0 | 32.1 |
| <O level | 30.1 | 18.7 | 17.8 | 18.3 | 15.4 | 14.7 | 11.4 | 11.0 |
| N sample | 12, 401 | 4,749 | 3,805 | 3,607 | 2,716 | 2,215 | 1,398 | 1,265 |
| **Paternal education** |  |  |  |  |  |  |  |  |
| A level or higher | 44.2 | 55.0 | 54.9 | 54.1 | 57.7 | 57.4 | 63.5 | 63.1 |
| O level | 21.3 | 21.3 | 21.2 | 21.6 | 20.8 | 21.2 | 20.8 | 20.4 |
| <O level | 34.6 | 23.7 | 23.9 | 24.3 | 21.5 | 21.4 | 15.7 | 16.5 |
| N sample | 11,923 | 4,654 | 3,722 | 3,543 | 2,677 | 2,178 | 1,385 | 1,255 |
| **Maternal Social Class** |  |  |  |  |  |  |  |  |
| High | 37.4 | 45.0 | 45.7 | 43.9 | 45.7 | 47.0 | 48.5 | 48.9 |
| Low | 62.7 | 55.0 | 54.3 | 56.1 | 54.4 | 53.0 | 51.5 | 51.1 |
| N sample | 10,047 | 4,147 | 3,368 | 3,136 | 2,381 | 1,965 | 1,398 | 1,265 |
| **Paternal Social Class** |  |  |  |  |  |  |  |  |
| High | 45.0 | 54.1 | 52.6 | 52.7 | 56.1 | 55.3 | 59.6 | 59.4 |
| Low | 55.0 | 45.9 | 47.4 | 47.3 | 43.9 | 44.7 | 40.4 | 40.7 |
| N sample | 10,937 | 4,390 | 3,523 | 3,341 | 2,525 | 2,073 | 1,372 | 1,240 |
| **Maternal Post-Partum depression** |  |  |  |  |  |  |  |  |
| No | 86.2 | 89.9 | 89.8 | 89.9 | 91.4 | 91.1 | 92.9 | 92.5 |
| Yes | 13.8 | 10.1 | 10.2 | 10.1 | 8.7 | 8.9 | 7.2 | 7.5 |
| N sample | 11,963 | 4,467 | 3,588 | 3,393 | 2,556 | 2,087 | 1,398 | 1,265 |
| **Physical Abuse 0-16y** |  |  |  |  |  |  |  |  |
| No | 85.1 | 83.0 | 82.8 | 79.1 | 79.5 | 79.5 | 80.3 | 79.6 |
| Yes | 14.9 | 17.0 | 17.2 | 20.9 | 20.5 | 20.5 | 19.7 | 20.4 |
| N sample | 6,440 | 3,886 | 3,018 | 3,096 | 2,493 | 1,992 | 1,398 | 1,265 |
| **Sexual Abuse 0-16y** |  |  |  |  |  |  |  |  |
| No | 97.2 | 96.0 | 96.2 | 94.4 | 94.3 | 94.6 | 95.6 | 96.1 |
| Yes | 2.8 | 4.0 | 3.8 | 5.6 | 5.7 | 5.4 | 4.4 | 3.9 |
| N sample | 9,112 | 4,583 | 3,595 | 3,510 | 2,703 | 2,181 | 1,398 | 1,265 |
| **Emotional Abuse 0-16y** |  |  |  |  |  |  |  |  |
| No | 80.7 | 81.1 | 81.3 | 80.2 | 81.3 | 80.6 | 83.1 | 81.9 |
| Yes | 19.3 | 18.9 | 18.7 | 19.8 | 18.8 | 19.4 | 16.9 | 18.1 |
| N sample | 6,914 | 3,968 | 3,091 | 3,062 | 2,459 | 1,972 | 1,398 | 1,265 |
| **Mother smoked in first 3 Months pregnancy** |  |  |  |  |  |  |  |  |
| No | 75.0 | 83.6 | 84.4 | 84.0 | 85.9 | 86.6 | 90.1 | 90.8 |
| Yes | 25.0 | 16.4 | 15.6 | 16.0 | 14.1 | 13.4 | 9.9 | 9.3 |
| N sample | 13,142 | 4,806 | 3,849 | 3,668 | 2,746 | 2,232 | 1,398 | 1,265 |
| **Early parenthood** |  |  |  |  |  |  |  |  |
| No | 91.9 | 96.3 | 96.3 | 96.2 | 96.9 | 96.7 | 98.3 | 98.3 |
| Yes | 8.1 | 3.7 | 3.7 | 3.8 | 3.1 | 3.3 | 1.7 | 1.7 |
| N sample | 13,960 | 4,875 | 3,920 | 3,728 | 2,781 | 2,261 | 1,398 | 1,265 |
