# Supplementary material for: Locus of Control and Negative Cognitive Styles in Adolescence as Risk Factors for Depression Onset in Young Adulthood: Findings From a Prospective Birth Cohort Study
Source: Front Psychol. 2021 Mar 25;12:599240. doi: 10.3389/fpsyg.2021.599240 (PMC8080877; doi:10.3389/fpsyg.2021.599240)
Supplement: Supplementary file 2 [file Table_2.docx]

Supplementary Material

Supplementary Table 2. Items of Nowicki-Strickland Internal-External scale (CNSIE) used to assess Locus of Control (LOC) at age 16.

| **Items CNSIE** | **Yes** | **No** |
| --- | --- | --- |
| E1. Do you feel that wishing can make good things happen? | 1 | 0 |
| E2. Are people nice to you no matter what you do? | 1 | 0 |
| E3. Do you usually do badly in your schoolwork even when you try hard? | 1 | 0 |
| E4. When a friend is angry with you is it hard to make that friend like you again? | 1 | 0 |
| E5. Are you surprised when your teacher praises you for your work in school? | 1 | 0 |
| E6. When bad things happen to you is it usually someone else's fault? | 1 | 0 |
| E7. Is doing well in your schoolwork just a matter of "luck" for you? | 1 | 0 |
| E8. Are you often blamed for things that just aren't your fault? | 1 | 0 |
| E9. When you get into an argument or fight is it usually the other person's fault? | 1 | 0 |
| E10. Do you think that preparing for things is a waste of time? | 1 | 0 |
| E11. When nice things happen to you is it usually because of "luck"? | 1 | 0 |
| E12. Does planning ahead make good things happen? | 0 | 1 |

The total scores are then added up to give a score ranging between 0 and 11 where higher scores indicate higher externality.
