# Supplementary material for: Locus of Control and Negative Cognitive Styles in Adolescence as Risk Factors for Depression Onset in Young Adulthood: Findings From a Prospective Birth Cohort Study
Source: Front Psychol. 2021 Mar 25;12:599240. doi: 10.3389/fpsyg.2021.599240 (PMC8080877; doi:10.3389/fpsyg.2021.599240)
Supplement: Supplementary file 3 [file Table_3.docx]

Supplementary Material

Supplementary Table 3. Items used in the Cognitive Styles Questionnaire (CSQ-SF) to assess negative cognitive styles at 17 years of age.

| **Items CSQ** |
| --- |
| **A. Imagine you are getting on badly with your parents** |
| A1: Agree/disagree: getting along badly with parents is caused by other people/circumstances |
| A2: Agree/disagree: reason for getting along badly with parents causes problems in all areas of life |
| A3: Agree/disagree: YP and parents will start fresh in the future and forget about the reasons for getting on badly |
| A4: Agree/disagree: getting along badly with parents means there is something wrong with YP as a person |
| A5: Agree/disagree: getting along badly with parents is YP's fault |
| A6: Agree/disagree: reason for getting along badly with parents does not stop YP enjoying things |
| A7: Agree/disagree: reason for getting along badly with parents will stop them getting along well in future |
| **B. Imagine your class reacts negatively to an important talk you have give as part of your coursework** |
| B1: Agree/disagree: class reacting badly to YP's talk is not YP's fault |
| B2: Agree/disagree: reason for class reacting badly to YP's talk will not cause failures in all areas of YP's life |
| B3: Agree/disagree: reason for class reacting badly to YP's talk will mean others will react negatively to YP's talks in the future |
| B4: Agree/disagree: class reacting badly to YP's talk says nothing about YP's strengths/weaknesses |
| B5: Agree/disagree: class reacting badly to YP's talk not caused by other people/circumstances |
| B6: Agree/disagree: reason for class reacting badly to YP's talk applies only to this talk |
| B7: Agree/disagree: reason for class reacting badly to YP's talk will not affect talks YP gives in the future |
| B8: Agree/disagree: class reacting badly to YP's talk means there is something wrong with YP as a person |
| **C. Imagine that during the first year of working in the career of your choice, you receive a megative evaluation of you job performance** |
| C1: Agree/disagree: negative evaluation in the first year of YP's chosen career would not be caused by other people/circumstances |
| C2: Agree/disagree: reason for negative evaluation in the first year of YP's chosen career would only apply to this judgement |
| C3: Agree/disagree: reason for negative evaluation in the first year of YP's chosen career would effect future job evaluations |
| C4: Agree/disagree: negative evaluation in the first year of YP's chosen career would mean there was something wrong with YP as a person |
| C5: Agree/disagree: negative evaluation in the first year of YP's chosen career would be YP's fault |
| C6: Agree/disagree: reason for negative evaluation in the first year of YP's chosen career would not cause failures in all areas of YP's life |
| C7: Agree/disagree: reason for negative evaluation in the first year of YP's chosen career would not impact future job evaluations |
| **D. Imagine you go to a party and people are not interested in you** |
| D1: Agree/disagree: people not being interested in YP at one party is YP's fault |
| D2: Agree/disagree: reason for people not being interested in YP at one party is specific to party |
| D3: Agree/disagree: if people are not interested in YP at one party then at future parties things will be different |
| D4: Agree/disagree: people not being interested in YP at a party means there is something wrong with YP as a person |
| D5: Agree/disagree: people not being interested in YP at a party was caused by other people/circumstances |
| D6: Agree/disagree: reason for people not being interested in YP at a party will cause problems in all areas of YP's life |
| D7: Agree/disagree: reason for people not being interested in YP at a party will cause people at future parties to be same |
| D8: Agree/disagree: people not being interested in YP at a party says a lot about YP as a person |
| **E. Imagine you receive a low mark for an exam** |
| E1: Agree/disagree: receiving a low mark in an exam is not YP's fault |
| E2: Agree/disagree: reason for receiving a low mark in an exam applies only to exa |
| E3: Agree/disagree: reason for receiving a low mark in an exam will cause YP to do badly in future exam |
| E4: Agree/disagree: receiving a low mark in an exam does not mean YP is flawed in some way |
| E5: Agree/disagree: receiving a low mark in an exam was not caused by other people/circumstances |
| E6: Agree/disagree: reason for receiving a low mark in an exam will not lead to failure in all areas of YP's life |
| E7: Agree/disagree: reason for receiving a low mark in an exam will not affect YP's performance in future exam |
| E8: Agree/disagree: receiving a low mark in an exam says a lot about YP as person |
| **F. Imagine you really want to be in an intimate, romantic relationship but you aren’t** |
| F1: Agree/disagree: not being in an intimate relationship is caused by other people/circumstances |
| F2: Agree/disagree: reason for not being in an intimate relationship is specific to intimate relationships |
| F3: Agree/disagree: reason for not being in an intimate relationship will have no effect on future relationships |
| F4: Agree/disagree: not being in an intimate relationship means there is something wrong with YP as a person |
| F5: Agree/disagree: not being in an intimate relationship is YP's fault |
| F6: Agree/disagree: reason for not being in an intimate relationship leads to problems in all areas of YP's life |
| F7: Agree/disagree: reason for not being in an intimate relationship means YP will not have intimate relationship in the future |
| F8: Agree/disagree: not being in an intimate relationship says nothing about YP as person |
| **G. Imagine that in an important class, you can’t complete all the work that your teacher expects of you** |
| G1: Agree/disagree: not being able to complete all the given work in an important class is not YP's fault |
| G2: Agree/disagree: reason for not being able to complete all the given work in an important class will cause problems in all areas of YP's life |
| G3: Agree/disagree: reason for not being able to complete all the given work in an important class will cause similar failures in completing future work |
| G4: Agree/disagree: not being able to complete all the given work in an important class says a lot about YP as a person |
| G5: Agree/disagree: not being able to complete all the given work in an important class was caused by other people/events |
| G6: Agree/disagree: reason for not being able to complete all the given work in an important class was specific to class |
| G7: Agree/disagree: reason for not being able to complete all the given work in an important class will not impact on completing work in future |
| G8: Agree/disagree: not being able to complete all the given work in an important class does not mean there is something wrong with YP as a person |
| **H. Imagine a person you’d really like to develop a close friendship with does not want to be friends with you** |
| H1: Agree/disagree: not being able to develop a close friendship with a specific person they like is caused by other people/circumstance |
| H2: Agree/disagree: reason for not being able to develop a close friendship with a specific person they like applies only to this friendship |
| H3: Agree/disagree: reason for not being able to develop a close friendship with a specific person they like will always prevent this person becoming YP's friend |
| H4: Agree/disagree: not being able to develop a close friendship with a specific person they like means there is something wrong with YP as a person |
| H5: Agree/disagree: not being able to develop a close friendship with a specific person they like is YP's fault |
| H6: Agree/disagree: reason for not being able to develop a close friendship with a specific person they like leads to problems in all areas of YP's life |
| H7: Agree/disagree: specific person they like may change mind and become YP's friend in the future |
| H8: Agree/disagree: not being able to develop a close friendship with a specific person they like says nothing about YP as a person |
| **I. Imagine you are unhappy** |
| I1: Agree/disagree: unhappiness is caused by other people/circumstances |
| I2: Agree/disagree: reason for unhappiness affects only YP's mood |
| I3: Agree/disagree: reason for unhappiness will always make YP unhappy |
| I4: Agree/disagree: unhappiness does not mean there is something wrong with YP as a person |
| I5: Agree/disagree: unhappiness is YP's fault |
| I6: Agree/disagree: reason for unhappiness causes problems in all areas of YP's life |
| I7: Agree/disagree: reason for unhappiness will go away and not cause YP to be unhappy in the future |
| I8: Agree/disagree: unhappiness says a lot about YP's strengths/weaknesses |

CSQtot, refers to total scores across all dimensions and scenarios, higher scores indicate more negative style Rowtotal of A1 A2 A3 A4 A5 A6 A7 A8 B1 B2 B3 B4 B5 B6 B7 B8 C1 C2 C3 C4 C5 C6 C7 C8 D1 D2 D3 D4 D5 D6 D7 D8 F1 F2 F3 F4 F5 F6 F7 F8 G1 G2 G3 G4 G5 G6 G7 G8 H1 H2 H3 H4 H5 H6 H7 H8 I1 I2 I3 I4 I5 I6 I7 I8) if CSQmiss ==0
