# Supplementary material for: Locus of Control and Negative Cognitive Styles in Adolescence as Risk Factors for Depression Onset in Young Adulthood: Findings From a Prospective Birth Cohort Study
Source: Front Psychol. 2021 Mar 25;12:599240. doi: 10.3389/fpsyg.2021.599240 (PMC8080877; doi:10.3389/fpsyg.2021.599240)
Supplement: Supplementary file 4 [file Table_4.docx]

Supplementary Material

Supplementary Table 4. Items of Short Mood and Feeling Questionnaire (SMFQ), completed at 23 years of age.

| **Question number** | **List of questions used SMFQ** | **Not True** | **Sometimes** | **True** |
| --- | --- | --- | --- | --- |
| 1 | I felt miserable or unhappy | 0 | 1 | 2 |
| 2 | I didn’t enjoy anything at all | 0 | 1 | 2 |
| 3 | I felt so tired I just sat around and did nothing | 0 | 1 | 2 |
| 4 | I was very restless | 0 | 1 | 2 |
| 5 | I felt I was no good anymore | 0 | 1 | 2 |
| 6 | I cried a lot | 0 | 1 | 2 |
| 7 | I found it hard to think properly or concentrate | 0 | 1 | 2 |
| 8 | I hated myself | 0 | 1 | 2 |
| 9 | I was a bad person | 0 | 1 | 2 |
| 10 | I felt lonely | 0 | 1 | 2 |
| 11 | I thought nobody really loved me | 0 | 1 | 2 |
| 12 | I thought I could never be as good as others | 0 | 1 | 2 |
| 13 | I did everything wrong | 0 | 1 | 2 |

The total scores are then added up to give a score ranging between 0 and 26 where higher scores indicate higher depressive symptoms. Questionnaire YPC: YPC1650, YPC1651, YPC1653, YPC1654, YPC1655, YPC1656, YPC1658, YPC1659, YPC1660, YPC1662, YPC166,3 YPC1665, YPC1667
