# Supplementary material for: Locus of Control and Negative Cognitive Styles in Adolescence as Risk Factors for Depression Onset in Young Adulthood: Findings From a Prospective Birth Cohort Study
Source: Front Psychol. 2021 Mar 25;12:599240. doi: 10.3389/fpsyg.2021.599240 (PMC8080877; doi:10.3389/fpsyg.2021.599240)
Supplement: Supplementary file 5 [file Table_5.docx]

Supplementary Material

Supplementary Table 5. Variables used to define ALSPAC-G1 parents in ALSPAC participants who did not enroll as parents in ALSPAC-G2

| Item | Variable | Age |
| --- | --- | --- |
| “YP has become a parent since the age of 12” | ccs2220 | 16+ |
| “YP is a parent” | YPA1000 | 21+ |
| “Respondent is a parent” | YPB7000 | 22+ |
| “Respondent is a parent (includes biological, step, foster and adopted children)” | YPC1050 | 23+ |
| “Respondent or partner is currently pregnant” | YPC1070 | 23+ |
| “YP is parent, biological, step, foster or adopted children” | YPD2000 | 24+ |
