# Supplementary material for: Locus of Control and Negative Cognitive Styles in Adolescence as Risk Factors for Depression Onset in Young Adulthood: Findings From a Prospective Birth Cohort Study
Source: Front Psychol. 2021 Mar 25;12:599240. doi: 10.3389/fpsyg.2021.599240 (PMC8080877; doi:10.3389/fpsyg.2021.599240)
Supplement: Supplementary file 6 [file Table_6.docx]

Supplementary Material

Supplementary Table 6. Variables used as exposures and confounding variables

| Variables | Variables’ names in ALSPAC | Age | Visit | Role in the model | Model |
| --- | --- | --- | --- | --- | --- |
| Locus of control (CNSIE) | (ccs3000 ccs3001 ccs3002 ccs3003 ccs3004 ccs3005 ccs3006 ccs3007 ccs3008 ccs3009 ccs3010 ccs3011) (z-score standardized) | 16+ | CCS | Exposure | Locus of control main model, mfq_t4 was used also as “previous” depression measure in NCS model. |
| Anxiety score (DAWBA) | Db15GAD (fh6500 fh6501 fh6502 fh6510 fh6511 fh6512 fh6513 fh6514 fh6515 fh6516 fh6517 fh6518 fh6519 fh6520 fh6530 fh6531 fh6540 fh6541 fh6542 fh6543 fh6544 fh6545 fh6546 fh6547 fh6548 fh6549 fh6550 fh6551 fh6570 fh6571 fh6572 fh6573 fh6574 fh6575) (z-score standardized) | 15+ | TF3 | Covariate (confounding factor) |  |
| Depressive symptoms scores (SMFQ) | mfq_t4 (ccs4500_ccs4502_ccs4503 ccs4504 ccs4505_ccs4506 ccs4508_ccs4509_ccs4511 ccs4512_ccs4513_ccs4514_rccs4515) (z-score standardized) | 16+ | CCS | Covariate (confounding factor) |  |
| Negative Cognitive styles (CSQ) | FJCQ1009 (z-score standardized) | 18 | F17 | Exposure | Negative cognitive styles main model |
| Anxiety score (CIS-R) | FJCI501 (z-score standardized) | 18 | F17 | Covariate (confounding factor) |  |
| Depressive symptoms scores (CIS-R) | FJCI1000 (z-score standardized) | 18 | F17 | Covariate (confounding factor) |  |
| Depressive symptoms scores (SMFQ) | fg7226 (fg7210-  fg7225) (z-score standardized) | 13+ | TF2 | Covariate (confounding factor) | Used in LOC model as “previous” depression measure |
| Sex | kz021 | Child baseline sample data |  | Covariate (confounding factor) | Confounding variables used in sensitivity analyses both in LOC and NCS models. |
| Physical abuse, sexual abuse, emotional abuse, emotional neglect, bullying, substance household, violence between parents, parental mental health problems or suicide, parent convicted offence, parental separation | ACEscore_classic_0_16yrs | 0-16 y | Classic ACEs | Covariate (confounding factor) |  |
| Maternal post-partum depression G0 (Edinburgh Post-natal Depression score) | b370 (z-score standardized) | 32 weeks of gestation | B | Covariate (confounding factor) |  |
| Maternal education | c645a recoded | 32 weeks of gestation | C | Covariate (confounding factor) |  |
| Maternal social class | c755 recoded | 32 weeks of gestation | C | Covariate (confounding factor) |  |
| Total IQ (WISC) | f8ws112 (z-score standardized) | 8+ | F8 | Covariate (confounding factor) |  |
