# Supplementary material for: Locus of Control and Negative Cognitive Styles in Adolescence as Risk Factors for Depression Onset in Young Adulthood: Findings From a Prospective Birth Cohort Study
Source: Front Psychol. 2021 Mar 25;12:599240. doi: 10.3389/fpsyg.2021.599240 (PMC8080877; doi:10.3389/fpsyg.2021.599240)
Supplement: Supplementary file 7 [file Table_7.docx]

Supplementary Material

Supplementary Table 7. Proportion of completed outcome, exposures and covariates across various samples

|  | **ALSPAC-G1 and G2 parents and non-parents** | | | | |
| --- | --- | --- | --- | --- | --- |
| **Measures** | **Parents and non-parents** | **Parents enrolled in ALSPAC-G2** | | **Only ALSPAC-G1 Parents** | **Parents G1 and G2 included in the analyses** |
|  | **Complete sample (N=14,872) (%)** | **ALSPAC-G2 parent sample (N=560) (%)** | **ALSPAC-G2 participants who have become parents before 293 months of age (24.4 years of age) (N=395) (%)** | **ALSPAC-G1 parent sample (N=307) (%)** | **ALSPAC-G1 and G2 participants who have become parents before 293 months of age (24.4 years of age) (N=790) (%)** |
| SMFQ N | 4,022 (27.0) | 385 (68.8) | 257 (65.1) | 168 (54.7) | 537 (68.0) |
| SMFQ+LOC N | 2,921 (19.6) | 245 (43.8) | 151 (38.2) | 93 (30.3) | 325 (41.1) |
| SMFQ+LOC+ COVARIATES N | 1,398 (9.4) | 97 (17.3) | 56 (14.2) | 24 (7.8) | 121 (15.3) |
| SMFQ+NCS N | 2,378 (15.9) | 202 (36.1) | 125 (31.6) | 60 (19.5) | 252 (31.9) |
| SMFQ+NCS+ COVARIATES N | 1,265 (8.5) | 80 (14.3) | 46 (11.6) | 17 (5.5) | 97 (12.3) |

Legend: COVARIATES: baseline depression and anxiety, SMFQ and DAWBA for the LOC model and CIS-R depression and anxiety subscale for NCS model, sex, maternal score of depression, maternal social class, maternal education, ACEs classic total score, IQ at 8 years old
