# Supplementary material for: Locus of Control and Negative Cognitive Styles in Adolescence as Risk Factors for Depression Onset in Young Adulthood: Findings From a Prospective Birth Cohort Study
Source: Front Psychol. 2021 Mar 25;12:599240. doi: 10.3389/fpsyg.2021.599240 (PMC8080877; doi:10.3389/fpsyg.2021.599240)
Supplement: Supplementary file 8 [file Table_8.docx]

Supplementary Material

Supplementary Table 8. Variables associated with Missingness in the Outcome and Exposures and in complete case record in ALSPAC Cohort (n= 14,872).

| Predictors of missingness^e^ | Odds of missingness in key variables | | | | | |  | |  | | |
| --- | --- | --- | --- | --- | --- | --- | --- | --- | --- | --- | --- |
|  | Depressive symptoms^a, b^ | | Locus of Control^b^ | | Negative Cognitive Styles^b^ | | Locus of Control complete model^c^ | | Negative Cognitive Styles complete model^d^ | | |
|  | OR | 95% CI, *p* | OR | 95% CI, *p* | OR | 95% CI, *p* | OR | 95% CI, *p* | OR | | 95% CI, *p* |
| Depressive symptoms SMFQ^a^ (n= 4,022) | _ | _ | 1.02 | 1.01 – 1.03, <0.001 | 1.01 | 1.00 – 1.02, 0.02 | 1.02 | 1.02 – 1.04, <0.001 | 1.02 | 1.01 – 1.03, <0.001 | |
| Locus of Control age 16 (n=5,106) | 1.15 | 1.09 – 1.22, <0.001 | _ | _ | 1.21 | 1.15 – 1.28, <0.001 | 1.26 | 1.18 – 1.34, <0.001 | 1.26 | 1.17 – 1.35, <0.001 | |
| Negative cognitive styles (n=4,173) | 1.01 | 0.94 – 1.07, 0.82 | 0.95 | 0.89 – 1.01, 0.12 | _ | _ | 0.96 | 0.90 – 1.03, 0.25 | 0.97 | 0.91 – 1.04, 0.38 | |
| Mother’s educational attainment (n=12,482) | 1.70 | 1.62 – 1.79, <0.001 | 1.86 | 1.78 – 1.95, <0.001 | 1.80 | 1.71 – 1.89, <0.001 | 2.13 | 1.97 – 2.30, <0.001 | 2.15 | 1.98 – 2.33, <0.001 | |
| Father’s educational attainment (n=12,000) | 1.46 | 1.40 – 1.53, <0.001 | 1.60 | 1.53 – 1.67, <0.001 | 1.51 | 1.44 – 1.58, <0.001 | 1.84 | 1.71 – 1.98, <0.001 | 1.79 | 1.66 – 1.93, <0.001 | |
| Maternal social class (10,109) | 1.49 | 1.37 – 1.63, <0.001 | 1.73 | 1.59 – 1.88, <0.001 | 1.70 | 1.56 – 1.85, <0.001 | 1.71 | 1.52 – 1.91, <0.001 | 1.73 | 1.53 – 1.94, <0.001 | |
| Paternal social class (11,006) | 1.57 | 1.44 – 1.70, <0.001 | 1.85 | 1.71 – 2.00, <0.001 | 1.58 | 1.45 – 1.71, <0.001 | 1.97 | 1.75 – 2.21, <0.001 | 1.92 | 1.71 – 2.17, <0.001 | |
| Mother smoked in pregnancy (13,348) | 2.12 | 1.92 – 2.34, <0.001 | 2.19 | 2.00 – 2.40, <0.001 | 2.23 | 2.02 – 2.46, <0.001 | 3.37 | 2.82 – 4.04, <0.001 | 3.60 | 2.97 – 4.38, <0.001 | |
| Mother’s early parenthood (14,189) | 2.67 | 2.23 – 3.20, <0.001 | 2.99 | 2.54 – 3.52, <0.001 | 2.82 | 2.36 – 3.38, <0.001 | 5.48 | 3.65 – 8.25, <0.001 | 5.36 | 3.50 – 8.21, <0.001 | |
| Mother’s post-partum EPDS score (12,150) | 1.24 | 1.19 – 1.29, <0.001 | 1.27 | 1.22 – 1.31, <0.001 | 1.22 | 1.17 – 1.27, <0.001 | 1.43 | 1.34 – 1.52, <0.001 | 1.37 | 1.29 – 1.47, <0.001 | |
| Financial difficulties 0-16y (7,629) | 1.39 | 1.21 – 1.59, <0.001 | 1.49 | 1.30 – 1.70, <0.001 | 1.54 | 1.34 – 1.77, <0.001 | 2.21 | 1.78 – 2.73, <0.001 | 2.10 | 1.69 – 2.61, <0.001 | |
| Parental separation 0-16y (6,603) | 1.21 | 1.08 – 1.36, 0.001 | 1.03 | 0.92 – 1.16, 0.57 | 1.34 | 1.19 – 1.49, <0.001 | 1.59 | 1.37 – 1.84, <0.001 | 1.56 | 1.34 – 1.82, <0.001 | |
| Physical abuse 0-16y (6,447) | 0.39 | 0.34 – 0.45, <0.001 | 0.65 | 0.56 – 0.75, <0.001 | 0.71 | 0.62 – 0.81, <0.001 | 0.64 | 0.55 – 0.74, <0.001 | 0.61 | 0.52 – 0.72, <0.001 | |
| Sexual abuse 0-16y (9,120) | 0.17 | 0.13 – 0.23, <0.001 | 0.41 | 0.31 – 0.53, <0.001 | 0.56 | 0.43 – 0.71, <0.001 | 0.56 | 0.42 – 0.75, <0.001 | 0.65 | 0.47 – 0.89, 0.007 | |
| Emotional abuse 0-16y (6,921) | 0.94 | 0.84 – 1.07, 0.35 | 1.06 | 0.94 – 1.20, 0.31 | 1.07 | 0.95 – 1.21, 0.27 | 1.22 | 1.05 – 1.43, 0.01 | 1.10 | 0.94 – 1.29, 0.23 | |
| Bullying 0-16y (7,071) | 1.04 | 0.93 – 1.16, 0.45 | 0.94 | 0.84 – 1.05, 0.25 | 0.85 | 0.76 – 0.95, 0.004 | 0.97 | 0.85 – 1.12, 0.71 | 0.98 | 0.85 – 1.13, 0.80 | |
| IQ 8 years (7,354) | 0.67 | 0.64 – 0.71, <0.001 | 0.63 | 0.60 – 0.66, <0.001 | 0.61 | 0.58 – 0.64, <0.001 | 0.59 | 0.56 – 0.63, <0.001 | 0.58 | 0.55 – 0.62, <0.001 | |
| Household substance abuse 0-16y (7,371) | 1.37 | 1.16 – 1.62, <0.001 | 1.34 | 1.15 – 1.57, <0.001 | 1.20 | 1.02 – 1.41, 0.03 | 1.62 | 1.29 – 2.03, <0.001 | 1.46 | 1.16 – 1.84, 0.001 | |
| Social class 0-16y (5,605) | 1.37 | 1.12 – 1.67, 0.002 | 1.49 | 1.24 – 1.79, <0.001 | 1.60 | 1.31 – 1.96, <0.001 | 2.01 | 1.44 – 2.81, <0.001 | 1.90 | 1.34 – 2.68, <0.001 | |
| Physical illness of the child 0-16y (9,292) | 1.46 | 1.25 – 1.71, <0.001 | 1.45 | 1.26 – 1.68, <0.001 | 1.32 | 1.13 – 1.53, <0.001 | 1.37 | 1.09 – 1.70, 0.005 | 1.54 | 1.21 – 1.96, <0.001 | |
| Violence between parents 0-16y (6,419) | 1.18 | 1.04 – 1.34, 0.01 | 1.33 | 1.18 – 1.51, <0.001 | 1.28 | 1.13 – 1.46, <0.001 | 1.32 | 1.12 – 1.55, 0.001 | 1.29 | 1.09 – 1.52, 0.002 | |
| Income (9,995) | 0.76 | 0.73 – 0.79, <0.001 | 0.68 | 0.65 – 0.71, <0.001 | 0.72 | 0.69 – 0.76, <0.001 | 0.64 | 0.60 – 0.67, <0.001 | 0.65 | 0.61 – 0.69, <0.001 | |
| BMI 17y (5,064) | 1.02 | 1.01 – 1.03, 0.01 | 1.04 | 1.03 – 1.06, <0.001 | 1.04 | 1.02 – 1.05, <0.001 | 1.05 | 1.03 – 1.06, <0.001 | 1.05 | 1.03 – 1.06, <0.001 | |

a: Depressive symptoms were assessed with SMFQ age 23.

b: Binary missingness markers were created as dummy variables, where 0 classified the variable as not missing and 1 as missing.

c: Binary missingness markers for the whole locus of control model were created: where 0 meant that Locus of control, SMFQ and all confounding factors were not missing, and 1 that they were missing

d: Binary missingness markers for the whole negative cognitive styles model were created: where 0 meant that Negative cognitive styles, SMFQ and all confounding factors were not missing, and 1 that they were missing

e: Predictor variables: Locus of control, negative cognitive styles and maternal EPDS score and IQ at 8 years of age were used as continuous standardized z-scores; mother’s and father’s educational attainment was coded as 0 = *A level or higher, 1 = O level, 2= < O level, maternal and paternal social class was coded as 0= high social class and 1= low social class, Mother smoked in pregnancy was coded 0= No and 1=Yes smoked in the first trimester of pregnancy cigarettes or cigars or other, Mother’s early parenthood (part of Family Adversity Index, the questionnaires was administered throughout the mother’s pregnancy: 8, 12, 18 and 32 weeks of gestation) was coded 0= risk factors was absent and 1=risk factor was present (i.e., age of first pregnancy/childbirth <20 years of age)*^1^*; Physical abuse, sexual abuse, emotional abuse, bullying, household substance abuse, social class, physical illness of the child and violence between parents were part of the Adverse Childhood Experiences (ACEs)*^2^ *and they were coded as 0=No and 1=Yes, income as G0 mothers weekly income at the time of their pregnancy, G1 BMI as measured at 17 years old.*
