# Supplementary material for: Locus of Control and Negative Cognitive Styles in Adolescence as Risk Factors for Depression Onset in Young Adulthood: Findings From a Prospective Birth Cohort Study
Source: Front Psychol. 2021 Mar 25;12:599240. doi: 10.3389/fpsyg.2021.599240 (PMC8080877; doi:10.3389/fpsyg.2021.599240)
Supplement: Supplementary file 9 [file Table_9.docx]

Supplementary Material

Supplementary Table 9. Variables used in multiple imputations.

| **Variables** | **Variable name** | **Values** | **Proportion of missingness compared to original sample *(N=14,872) (%)*** |
| --- | --- | --- | --- |
| Depressive symptoms as measured with Short Mood and Feeling Questionnaire at 23+ years of age | mfq_t9 | 0-26 | 27% |
| Locus of control measured with Nowicki-Strickland Internal-External scale (CNSIE) at age 16+ | Locus_Control_rc | 0-11 (range in data) | 34% |
| Negative cognitive styles measured with Short-Cognitive Styles Questionnaire at 17+ | FJCQ1009 | 92-273 (range in data) | 28% |
| **Confounders** | **Variable name** | **Values** |  |
| Sum of 5 depressive scores measured with CIS-R at 17 + years of age | FJCI1000 | 0-21 (range in data) | 31% |
| Anxiety score measured with CIS-R at 17 + years of age | FJCI501 | 0-4 (range in data) | 31% |
| Generalised anxiety disorder assessed with DAWBA section C at 15+ | Db15GAD | 0-40 (range in data) | 36% |
| Depressive symptoms as measured with Edinburgh Post-natal Depression Score in mothers G0 during pregnancy | b370 | 0-30 (range in data) | 82% |
| Maternal social class | mat_sclass | 0=high social class  1=low social class | 68% |
| IQ measured with WISC at 8 years of age | f8ws112 | 45-151 (range in data) | 49% |
| Maternal educational | mat_edu | 0 = A level or higher  1 = O level  2 = <O level/CSE/vocational | 84% |
| Child sex | sex_rc | 0=male  1=female | 100% |
| **Auxiliary variables** | **Variables name** | **Values** |  |
| Depressive symptoms as measured with Short Mood and Feeling Questionnaire at 10 years of age | mfq_t1 | 0-23 (range in data) | 50% |
| Depressive symptoms as measured with Short Mood and Feeling Questionnaire at 12+ years of age | mfq_t2 | 0-25 (range in data) | 45% |
| Depressive symptoms as measured with Short Mood and Feeling Questionnaire at 13+ years of age | mfq_t3 | 0-26 | 41% |
| Depressive symptoms as measured with Short Mood and Feeling Questionnaire at 16+ years of age | mfq_t4 | 0-26 | 34% |
| Depressive symptoms as measured with Short Mood and Feeling Questionnaire at 17+ years of age | mfq_t5 | 0-26 | 30% |
| Depressive symptoms as measured with Short Mood and Feeling Questionnaire at 18+ years of age | mfq_t6 | 0-26 | 22% |
| Depressive symptoms as measured with Short Mood and Feeling Questionnaire at 21+ years of age | mfq_t7 | 0-26 | 22% |
| Depressive symptoms as measured with Short Mood and Feeling Questionnaire at 22+ years of age | mfq_t8 | 0-26 | 26% |
| Paternal educational | pat_edu | 0 = A level or higher  1 = O level  2 = <O level/CSE/vocational | 81% |
| Locus of control measured with Nowicki-Strickland Internal-External scale (CNSIE) at age 8+ | f8lc125 | 0-12 (range in data) | 43% |
| Paternal social class | pat_sclass | 0=high social class  1=low social class | 74% |
| Average income weekly at 33and 47 months | avinc | 75.90 - 806.65 (range in data) | 67% |
| Total score of Classic Adverse Childhood Experiences | z_ACEscore_classic_0_16yrs | 0-10 (range in data) | 24% |
| Depressive symptoms in parents enrolled in alspac-g2 measured with EPDS | depression_parent | 0-24 (range in data) | 2.0% |
| Any anxiety disorder (self-report 6-band computer prediction, ICD-10 and DSM-IV) at age 15+ | fh6877 | 0-5 (range in data) | 36% |
| Anxiety Sensitivity Index Physical concerns score at age 17+ | FJLE220 | 10-50 (range in data) | 29% |
| Anxiety Sensitivity Index Mental concerns score at age 17+ | FJLE221 | 8-40 (range in data) | 30% |
| Crown Crisp Anxiety Score mother G0 | b351 | 0-16 (range in data) | 80% |
| IQ measured with WISC at 13 years of age | fh6280 | 55-137 (range in data) | 33% |
| Depression (self-report 6-band computer prediction, ICD-10 and DSM-IV) at age 15+ | fh6876 | 0-5 (range in data) | 36% |
| Mother smoked during 1^st^ trimester of pregnancy | b665 | 0=no  1=yes | 90% |
