# Supplementary material for: Locus of Control and Negative Cognitive Styles in Adolescence as Risk Factors for Depression Onset in Young Adulthood: Findings From a Prospective Birth Cohort Study
Source: Front Psychol. 2021 Mar 25;12:599240. doi: 10.3389/fpsyg.2021.599240 (PMC8080877; doi:10.3389/fpsyg.2021.599240)
Supplement: Supplementary file 10 [file Table_10.docx]

Supplementary Material

Supplementary Table 10. Main analyses in complete case sample.

|  |  | | |  |  |  |  | | Locus of Control | | | | | |  | | | Negative Cognitive Styles | | |  | | | | | | |  | | | | |
| --- | --- | --- | --- | --- | --- | --- | --- | --- | --- | --- | --- | --- | --- | --- | --- | --- | --- | --- | --- | --- | --- | --- | --- | --- | --- | --- | --- | --- | --- | --- | --- | --- |
| Sample | **Unadjusted complete case^a^** | | | | **Adjusted^b^** | | **Unadjusted complete case^c^** | | | | | | **Adjusted^d^** | | **Unadjusted complete case^a^** | | | | **Adjusted^b^** | | **Unadjusted complete case^c^** | | | | | | | **Adjusted^d^** | | | | |
|  | N | OR (95% CI) | *P* Value | | OR (95% CI) | *P* Value | N | OR (95% CI) | | *P* Value | | OR (95% CI) | | *P* Value | N | OR (95% CI) | *P* Value | | OR (95% CI) | *P* Value | N | OR (95% CI) | | *P* Value | | | OR (95% CI) | | | *P* Value |  |  |
| Total sample | 1,398 | 1.61 (1.40 – 1.84) | <0.001 | | 1.12 (0.95 – 1.32) | 0.17 | 1,371 | 1.60 (1.40 – 1.84) | | | 0.001 | 1.31 (1.12 – 1.52) | | <0.001 | 1,265 | 1.44 (1.26 – 1.66) | <0.001 | | 1.24 (1.07 – 1.44) | 0.005 | 1,124 | | 1.46 (1.26 – 1.69) | | <0.001 | | 1.17 (0.99 – 1.38) | | 0.06 | | |  |
| Parents | 121 | 2.80 (1.74 – 4.50) | <0.001 | | 2.54 (1.38 – 4.70) | 0.003 | 117 | 2.70 (1.68 – 4.36) | | <0.001 | | 2.46 (1.36 – 4.46) | | 0.003 | 97 | 1.22 (0.81 – 1.85) | 0.34 | | 1.09 (0.60 – 1.98) | 0.78 | 86 | | 1.25 (0.80 – 1.94) | | | 0.33 | 1.04 (0.48 – 2.22) | | | 0.93^[[1]](#footnote-1)^ | |  |

1. Unadjusted and adjusted logistic regressions with binary SFMQ at age 23 as outcome variable.

   ^a^ Unadjusted complete case regressions between LOC or NCS on binary score of depression. Sample was restricted to those who has information on outcome, exposure, and all confounding factors (concurrent depression and demographic and life event variables)

   ^b^ Adjusted logistic regression, confounding factors were: concurrent depression, anxiety, sex, maternal education, social class and depression score, child IQ at 8 years of age.

   ^c^ Unadjusted complete case regressions between LOC or NCS on binary score of depression. Sample was restricted to those who has information on outcome, exposure, and all confounding factors (previous depression and demographic and life event variables)

   ^d^ Adjusted logistic regression, confounding factors were: previous depression, anxiety, sex, maternal education, social class and depression score, child IQ at 8 years of age. [↑](#footnote-ref-1)
