# Supplementary material for: Locus of Control and Negative Cognitive Styles in Adolescence as Risk Factors for Depression Onset in Young Adulthood: Findings From a Prospective Birth Cohort Study
Source: Front Psychol. 2021 Mar 25;12:599240. doi: 10.3389/fpsyg.2021.599240 (PMC8080877; doi:10.3389/fpsyg.2021.599240)
Supplement: Supplementary file 11 [file Table_11.docx]

Supplementary Material

Supplementary Table 11. Complete case: Unadjusted and Adjusted Odds Ratio for Adult Depression According to continuous scores of Locus of Control and Stratified by Parental Status.

|  | Moderation model by parenthood | | | | | |  | |
| --- | --- | --- | --- | --- | --- | --- | --- | --- |
|  | Entire sample  (1,398) | | Parents^a^  (121) | | Non-parents  (1,277) | | Interaction term  (1,398) | |
|  | OR | 95% CI, *p* | OR | 95% CI, *p* | OR | 95% CI, *p* | OR | 95% CI, *p* |
| Locus of control | 1.61 | 1.40 – 1.84, <0.001 | 2.80 | 1.74 – 4.50, <0.001 | 1.51 | 1.30 – 1.74, <0.001 | 1.86 | 1.13 – 3.04, 0.02 |
| Locus of control adjusted for all confounding factors | 1.12 | 0.95 – 1.31, 0.17 | 2.54 | 1.38 – 4.70, 0.003 | 1.05 | 0.88 – 1.25, 0.60 | 1.87 | 1.10 – 3.19, 0.21 |

Outcome: binary SMFQ

a: all ALSPAC parents (regardless their enrolment in ALSPAC-G2)
