# Supplementary material for: Locus of Control and Negative Cognitive Styles in Adolescence as Risk Factors for Depression Onset in Young Adulthood: Findings From a Prospective Birth Cohort Study
Source: Front Psychol. 2021 Mar 25;12:599240. doi: 10.3389/fpsyg.2021.599240 (PMC8080877; doi:10.3389/fpsyg.2021.599240)
Supplement: Supplementary file 12 [file Table_12.docx]

Supplementary Material

Supplementary Table 12. Complete case: Unadjusted and Adjusted Odds Ratio for Adult Depression According to continuous scores of Cognitive Negative Styles and Stratified by Parental Status.

|  | Moderation model by parenthood | | | | | |  | |
| --- | --- | --- | --- | --- | --- | --- | --- | --- |
|  | Entire sample  (1,265) | | Parents^a^  (97) | | Non-parents  (1,168) | | Interaction term  (1,265) | |
|  | OR | 95% CI, *p* | OR | 95% CI, *p* | OR | 95% CI, *p* | OR | 95% CI, *p* |
| Cognitive Styles | 1.44 | 1.26 – 1.66, <0.001 | 1.22 | 0.81 – 1.85, 0.34 | 1.47 | 1.28 – 1.70, <0.001 | 0.83 | 0.66 – 1.83, 0.72 |
| Cognitive styles adjusted for baseline depression and anxiety | 1.24 | 1.07 – 1.44, 0.005 | 1.09 | 0.60 – 1.98, 0.78 | 1.27 | 1.08 – 1.49, 0.003 | 0.72 | 0.45 – 1.15, 0.17 |

Outcome: binary SMFQ

a: all ALSPAC parents (regardless their enrolment in ALSPAC-G2)
