# Supplementary material for: Locus of Control and Negative Cognitive Styles in Adolescence as Risk Factors for Depression Onset in Young Adulthood: Findings From a Prospective Birth Cohort Study
Source: Front Psychol. 2021 Mar 25;12:599240. doi: 10.3389/fpsyg.2021.599240 (PMC8080877; doi:10.3389/fpsyg.2021.599240)
Supplement: Supplementary file 13 [file Table_13.docx]

Supplementary Material

Supplementary Table 13. Complete case: Unadjusted and Adjusted Odds Ratio for Adult Depression According to continuous scores of Locus of Control and Stratified by Sex.

|  | Analyses stratified by sex | | | | | |
| --- | --- | --- | --- | --- | --- | --- |
|  | Entire sample  (1,398) | | Male  (517) | | Female  (881) | |
|  | OR | 95% CI, *p* | OR | 95% CI, *p* | OR | 95% CI, *p* |
| Locus of control | 1.61 | 1.40 – 1.84, <0.001 | 1.33 | 1.03 – 1.70, 0.03 | 1.72 | 1.46 – 2.03, <0.001 |
| Locus of control adjusted for baseline depression and anxiety | 1.12 | 0.95 – 1.31, 0.17 | 0.90 | 0.66 – 1.23, 0.50 | 1.23 | 1.01 – 1.50, 0.04 |

Outcome: binary SMFQ
