# Supplementary material for: Locus of Control and Negative Cognitive Styles in Adolescence as Risk Factors for Depression Onset in Young Adulthood: Findings From a Prospective Birth Cohort Study
Source: Front Psychol. 2021 Mar 25;12:599240. doi: 10.3389/fpsyg.2021.599240 (PMC8080877; doi:10.3389/fpsyg.2021.599240)
Supplement: Supplementary file 14 [file Table_14.docx]

Supplementary Material

Supplementary Table 14. Complete case: Unadjusted and Adjusted Odds Ratio for Adult Depression According to continuous scores of Cognitive Negative Styles and Stratified by Sex.

|  | Analyses Stratified by sex | | | | | |
| --- | --- | --- | --- | --- | --- | --- |
|  | Entire sample  (1,265) | | Male  (452) | | Female  (813) | |
|  | OR | 95% CI, *p* | OR | 95% CI, *p* | OR | 95% CI, *p* |
| Cognitive Styles | 1.44 | 1.26 – 1.66, <0.001 | 1.61 | 1.23 – 2.11, 0.001 | 1.39 | 1.19 – 1.62, <0.001 |
| Cognitive styles adjusted for baseline depression and anxiety | 1.24 | 1.07 – 1.44, 0.005 | 1.38 | 1.02 – 1.85, 0.04 | 1.21 | 1.02 – 1.44, 0.03 |

Outcome: binary SMFQ
